# Green Extraction of Natural Antioxidants from Agro-Food and Marine Bioresources Using Vegetable Oils

**DOI:** 10.3390/antiox15070875

**Published:** 2026-07-14

**Authors:** Adriana Slavova-Kazakova, Svetlana Momchilova

**Affiliations:** 1 Laboratory of Lipid Chemistry, Institute of Organic Chemistry with Centre of Phytochemistry, Bulgarian Academy of Sciences, Acad. Georgi Bonchev Str., Block 9, 1113 Sofia, Bulgaria; 2 Centre of Competence “Sustainable Utilization of Bio-Resources and Waste of Medicinal and Aromatic Plants for Innovative Bioactive Products” (BIORESOURCES BG), 1000 Sofia, Bulgaria

**Keywords:** vegetable oils, green solvents, lipid soluble biomolecules, antioxidants

## Abstract

This review intends to provide an insight into the wide range of possibilities for vegetable oils to be used as solvents to extract natural ingredients for various applications with a special emphasis on antioxidants. The potential of using oils as food-grade solvents for extraction of carotenoids, crocins, curcuminoids, cannabinoids, capsaicinoids, different volatile organic compounds and other lipid-soluble phytochemicals from plant and marine sources and by-products is summarized. Most studies focus on optimizing extraction parameters and evaluating the physical and chemical characteristics of the obtained oily plant extract. On the one hand, these infused or enriched oils can be considered as plant extracts, but on the other hand, one should not ignore the fact that lipid oxidation is a problem that needs to be addressed. The characterization and analysis of the obtained oily extracts is closely related to their specific application in the food or cosmetic industry. Despite all the advantages, disadvantages related to the stability of the fortified oils are discussed as well.

## 1. Introduction

**Scheme 1:**
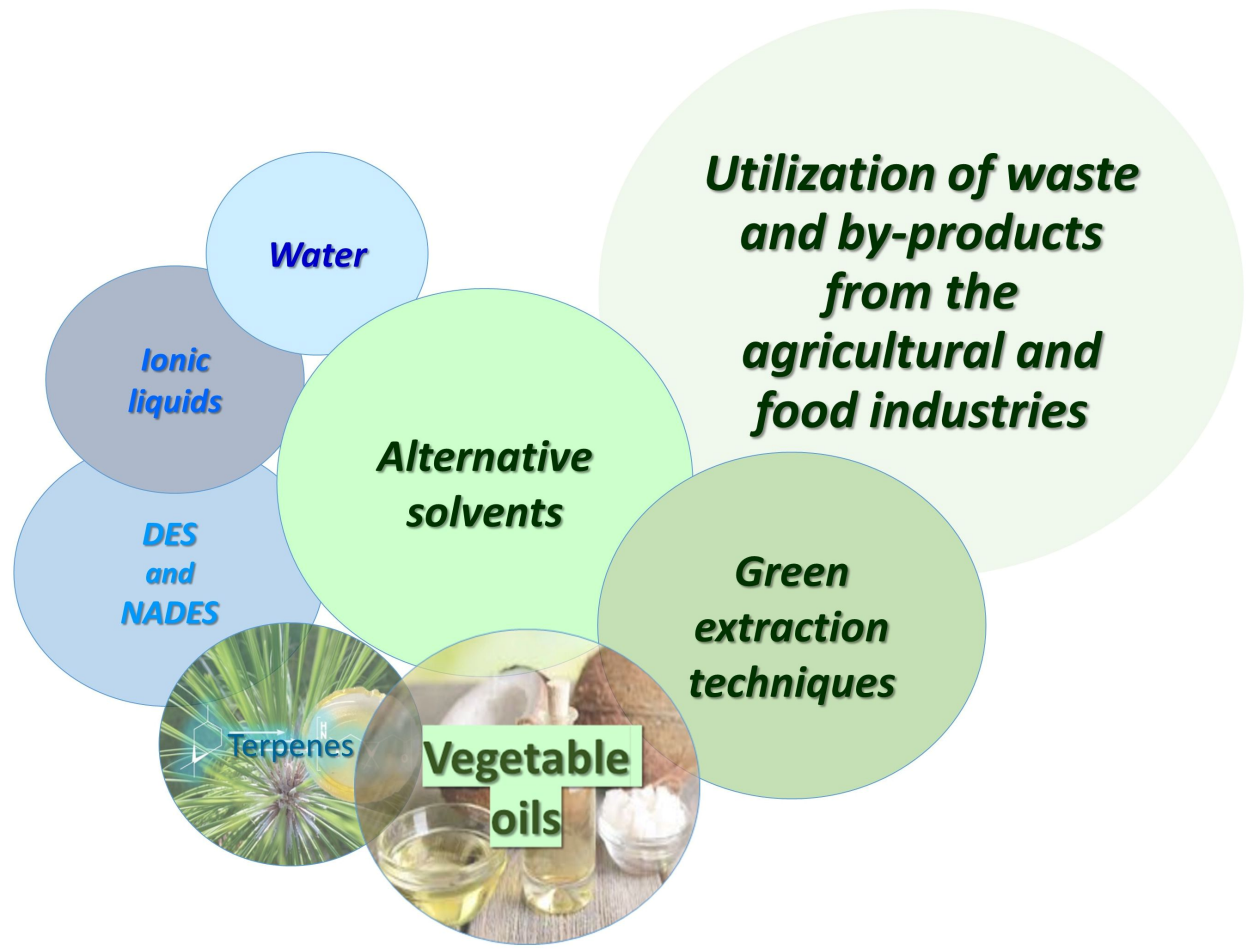
Green chemistry: state-of-the-art.

The main challenges facing the circular economy as a vital tool for achieving the Sustainable Development Goals (SDGs) are related not only to actual use of waste and by-products from the agricultural industry, but also to find specific, low-cost and efficient methodologies for using and converting the biomass into reagents, ingredients and products. One of the 12 principles of the green chemistry concept, developed by Anastas and Warner in 1998, requires application of green and less hazardous solvents [1]. The reduction of organic solvents’ usage is one of the most important goals of the transition from a petroleum-based economy to a more environmentally friendly and sustainable one. Replacing them with renewable and non-hazardous options makes the extraction procedure much safer. Supercritical and subcritical fluids, ionic liquids, deep eutectic solvents and those derived from renewable sources (Scheme 1) are among the proposed green alternatives to commonly used petrochemical solvents [2,3,4]. Vegetable oils, obtained from plant renewable sources, are non-toxic, non-volatile, non-irritative and have a relatively high flash point and a selective dissolving power [5]. Edible oils also have the advantage that they could be directly incorporated into food and cosmetic products [6]. Furthermore, their use in extraction processes would be economically much more advantageous compared not only to the conventional organic solvents, but also to most green alternatives listed above.

The nature of the plant material and the target compounds are decisive for the choice of solvent as well as the extraction method in order to achieve a maximal extraction efficiency [2]. Thus, methanol demonstrates superior capacity for the total extraction of phenolic compounds from plant matrices, particularly for lower molecular weight polyphenols, including phenolic acids and flavonoid glycosides, compared to ethanol or water–ethanolic solutions. However, water–ethanolic mixtures (usually 50–80% ethanol) are often preferred for food-grade and safe applications due to methanol’s high toxicity. Water could be an excellent option for hydrophilic compounds such as proteins, peptides and polysaccharides, whereas the finding of more environmentally friendly substitutes of dichloromethane, ether or hexane in the extraction of lipophilic compounds is a more complex task. The latter also aims to overcome the obstacles and reduce the costs associated with purification steps after solvent evaporation [7]. After pre-treatment of the plant material (biomass) and the extraction itself, the following steps/stages are necessary and unavoidable when using organic solvents: solid–liquid separation, solvent evaporation from both the final extract and spent plant residue, and finally, solvent purification (distillation) [3]. In this regard, using water could result in high energetic demands during the post-extraction stages required for its removal. On the other hand, applying edible vegetable oils for the extraction of lipid soluble phytochemicals from plant matrices could result in ready-to-use extracts whose valuable physical, chemical and/or biological properties determine their suitability for inclusion in the composition of a final product [6]. However, there are some disadvantages, too, related to oxidative changes in the physical and chemical properties of the oils, which are one of the biggest obstacles in extraction processes.

Historically, obtaining infused oils using medicinal and aromatic plants dates back to ancient times, but this idea began to attract the attention of the scientific community rather late. Scientific research on oils for the preparation of ready-to-use extracts for food and cosmetic application became attractive again with the development of the topic of agricultural waste products valorization. The combination of green solvents as a non-toxic alternative to organic solvents with state-of-the art extraction techniques reveals a significant potential for the future of sustainable industries.

Here we attempt to organize and build on our findings from the literature survey by taking as a basis and framework the target molecules that were reviewed in two main categories of lipophilic compounds., i.e., non-phenolic and phenolic. Such representatives of the former are mainly carotenoids, while the phenol group includes terpenophenolic cannabinoids, as well as some alkaloids (capsaicinoids) possessing phenolic groups.

## 2. Literature Search and Data Collection

This review aligns with the state-of-the-art green extraction of natural antioxidants from plant sources and by-products, using vegetable oils.

Scientific papers were collected from databases of Scopus and Web of Science using the key words “vegetable oils as green solvents”, “plant-based oils as alternative solvents”, “extraction of lipid soluble compounds (phytochemicals) with vegetable oils”, “infused oils”, “enriched oils”, “functional oils”. The articles (more than 3650, published mainly by the end of 2025) were screened for the information they contained in order to achieve the aim of this review. After removing the duplicate records, only those scientific articles were reviewed in which plant-based oils (including pure vegetable oils, but not essential oils) were evaluated as green solvents alternative to organic ones. The published studies on the extraction of plant oils themselves from various natural sources, even if by green extraction methods, were also excluded. In addition, although a body of scientific literature concerns the olive processing and especially the oil-based extraction of phytochemicals from side streams and by-products, they left out of the focus of this review. To assess the potential of vegetable oils as green solvents, the following data were summarized (including two tables): type of natural source from which the target compounds were extracted, the extraction procedure(s) and conditions, functional properties and/or activities of the extracts obtained. Our second goal, in line with the topic of the journal, was to emphasize the antioxidant activity of the groups of compounds presented here, and for that reason, a brief information on the mechanism of action of the representatives of these groups was given as well.

## 3. Bioactive Compounds with Antioxidant Properties Extracted by Vegetable Oils

In this review, along with the classical extraction techniques such as maceration and infusion, some intensification procedures including ultrasound or microwaves treatment are presented and summarized as well. The focus is on the following extracted bioactive compounds with antioxidant properties: carotenoids, crocins, curcuminoids, cannabinoids, capsaicinoids and fractions from medicinal/aromatic plants, obtained by extraction using vegetable oils.

### 3.1. Carotenoids

**Figure 1:**
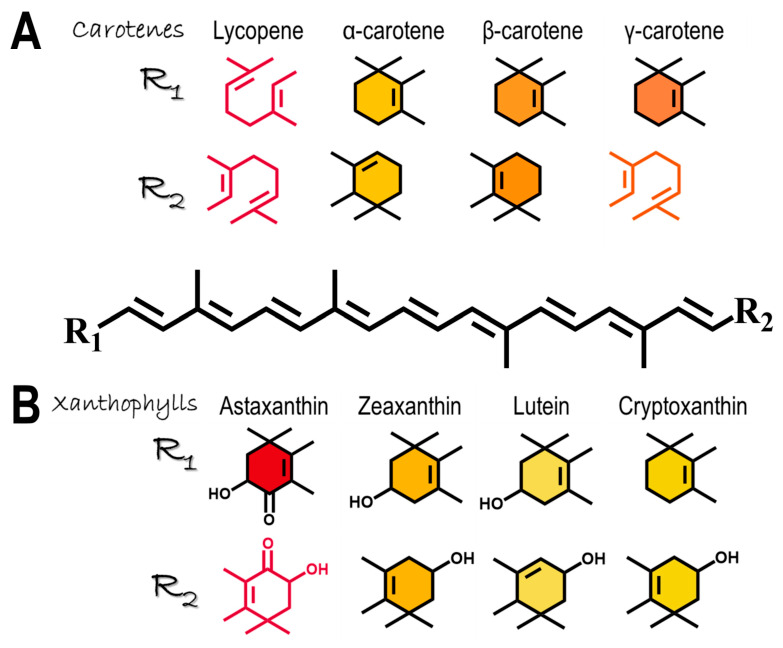
Chemical structures of the main carotenoid representatives of (**A**) carotenes; (**B**) xanthophylls.

Carotenoids are the most widespread natural pigments, with over 600 isolated substances. In terms of their chemical structure, they are divided in two main classes: (i) carotenes, which are polyunsaturated hydrocarbons (Figure 1A), and (ii) xanthophylls, which contain oxygen atoms, mostly in hydroxyl groups (Figure 1B).

All carotenoids are capable of acting as singlet oxygen quenchers, with efficiency depending on their molecular structure. It should be pointed out that the natural sensitizers in plant oils, e.g., the chlorophylls, may generate singlet oxygen during oil oxidation under light. The stabilizing effects of carotenoids are due to their ability to quench the singlet oxygen, which explains their cooperative (synergistic) action with co-antioxidants, especially vitamins E (tocopherols) and C.

Despite the large number of carotenoids, only about 50 of them are precursors of vitamin A (the so-called provitamin A) with beta-carotene being the most abundant carotenoid in human diet. The antioxidative behavior of beta-carotene is closely related to its own oxidation. The rate and mechanism of that oxidative degradation at different conditions are reviewed by Yanishlieva et al. [8]. When broken down in the human body (in the small intestine), beta-carotene yields two retinal molecules, which subsequently are reduced to retinol, while the cleavage of alpha- and gamma-carotene results in only one retinal molecule. The antioxidant mechanism of retinol is linked to its interaction with thiol-containing metabolites, helping to maintain, for example, glutathione (GSH) in its reduced form relative to its oxidized form (GSSG). Retinol reacts with thiyl-free radicals, formed when GSH scavenges free radicals, to form a stable radical, thereby stopping chain-reaction damage and thus exerting an antioxidant effect.

Although popular carotenoids such as lycopene, lutein, zeaxanthin and astaxanthin (Figure 1) are non-provitamin A forms they have been demonstrated to be potent antioxidants. Moreover, they are associated with a reduced risk of infectious diseases and have shown potential protective roles against inflammatory conditions, cardiovascular and neurodegenerative disorders, certain types of cancer, diabetic complications, age-related macular degeneration, liver diseases, etc. [9,10,11].

Due to their valuable properties, including antioxidant effects, carotenoids are target compounds for extraction and further processing in food, nutraceutical, pharmaceutical and cosmetic industries.

#### 3.1.1. Total Carotenoids

The most widespread and rich source of carotenoids is the carrot (*Daucus carota* L.), grown as an edible root worldwide. Its chemical composition, especially the amount and proportions of bioactive compounds, depends on the genotype, cultivation method, as well as on the soil and climatic conditions. Usually, carotenoids are extracted as a total fraction but if the plant source contains predominantly a specific target compound the technological processing could be optimized to enable a recovery of such enriched extract.

Extraction of carotenoids from dried carrots with refined soybean oil and extra virgin olive oil was carried out by Borguini et al. [12] (details in Table 1) in order to enrich these oils with provitamin A forms. The extraction conditions were optimized applying the Central Composite Rotational Design (CCRD) of Response Surface Methodology (RSM) in regard to the yield of total carotenoids, lutein, alpha- and beta-carotenes, depending on both the ratio of plant material to oil volume, and the extraction time. The authors concluded that 20% of the plant matrix (dried carrot) in vegetable oil and 5 min extraction time was the optimal combination for soybean and extra virgin olive oils. Moreover, a dose of 10 mL of these enriched oils could contribute to meet the Recommended Daily Intake for vitamin A (600 μg retinol equivalent) in the human diet.

Dried carrots as a plant source of carotenoids were extracted also by sunflower oil as an alternative to the commonly used organic solvents such as hexane [13] (Table 2). The procedure was optimized by applying an ultrasound intensification technique, which resulted in a threefold increase in beta-carotene concentration with an increase of the ratio “dried plant material to oil” from 1:10 to 2:10, but without further raising above 3:10. The authors chose 35 min as the maximum time limit in RSM optimization, considering that 30 min was a sufficient time to achieve 80% extraction yield.

A valuable by-product in the carrot juice manufacturing is the carrot pomace, and therefore it is reasonable and worth paying attention to its utilization. Thus, the Response Surface Methodology was applied by Han et al. [14] (Table 2) to optimize carotenoids extraction from carrot processing waste, using cotton seed oil as a solvent, with ultrasonic intensification of the process. The authors determined optimal recovery of 80.3% at 213 W ultrasonic power, extraction time of 36.0 min and temperature of 44.6 °C.

The same optimization approach, i.e., RSM, was employed also by Elik et al. to evaluate the effects on carotenoids recovery of the microwave power, extraction time, and ratio between the plant material and solvent, when carrot waste was extracted by flaxseed oil [15] (Table 2). The following optimal conditions for 77.48% recovery were established: 165 W microwave power, 9.39 min extraction time and ratio of 8.06:1 g waste/g oil.

The results published in the literature cannot be compared due to various research objects used (dried vs. fresh carrots and their processing residues), extracted by different methods with several types of vegetable oils as solvents, sometimes assisted by ultrasound or microwaves at different conditions, etc.

In addition to the carrot juice manufacture, there are other juice-processing industries that generate plenty of waste that can be utilized as valuable by-products, containing a plethora of bioactive components such as carotenoids.

*Bactris gasipaes* (peach palm) is a tropical tree cultivated for its edible nutritious fruits consumed since pre-Colombian time, and nowadays is used to produce food products such as flour, jams, chips and fermented beverages. A study published by Ordoñez-Santos and co-workers [16] (Table 2) aimed to evaluate the effect of extraction time, solid to solvent ratio, and temperature that affected ultrasonic-assisted extraction (UAE) of carotenoids from dry peach palm peel using soybean oil as a green solvent. Optimization of the conditions for maximal total carotenoids extraction from dried peels was carried out applying Central Composite Design of RSM. The optimal conditions for UAE were 48 °C, extraction time of 28 min, and solid–solvent ratio of 0.0037 g/mL, with a predicted carotenoid content of 151.50 mg/100 g dry peels. UAE was compared with the Soxhlet extraction and maceration as conventional methods ensuring carotenoids yield of 168.8 ± 3.50 and 113.40 ± 1.20 mg/100 g dried peel, respectively.

Carotenoids were also extracted from mango pulp by groundnut, sunflower and flaxseed oils [17] (Table 1 and Table 2). Different extraction techniques were employed including magnetic stirring, ultrasonication, microwave and high shear dispersion for different time intervals at different pulp to vegetable oil ratios. Thereby, 540 samples were analyzed but one combination was selected on the basis of the highest total carotenoid content using split plot design of statistical analysis.

In another study, three types of green solvents (vegetable oils, fatty acids and deep eutectic solvents) were compared in terms of carotenoids extraction and antioxidant activity of orange peel [18] (Table 2). Regarding the olive oil tested, its extract exhibited a very high antioxidant activity assessed by ABTS and FRAP methods, which could be attributed to the inherent antioxidant activity of the oil itself.

Based on UAE of carotenoids from pomegranate peels, using sunflower and soybean oils as solvents, Goula et al. [19] (Table 2) investigated the extraction time, solid/oil ratio, amplitude level and temperature with respect to the extraction yield. The authors found that a time period of 30 min was sufficient to achieve maximum carotenoid yield. The optimum operating conditions were extraction temperature 51.5 °C, peels/solvent ratio of 0.10 and amplitude level 58.8% with sunflower oil as a solvent. A second-order kinetic model was successfully developed for describing the mechanism of ultrasound extraction under different processing parameters.

Evaluation of the efficiency of three different extraction systems (oil-in-water O/W emulsions, oils and organic solvents) on the yields of carotenoids and beta-sitosterol extracted from buckthorn pomace was performed by Taarji and co-workers [20] (Table 2). Compared with six organic solvents (acetone, chloroform, ethyl acetate, hexane, 50% and 99.5% ethanol) and triacylglycerols of two refined oils (soybean and rapeseed oil), O/W emulsions prepared without emulsifier using a high-pressure homogenizer had the highest extraction capability for β-sitosterol and carotenoids.

The optimization of various parameters (time, temperature, ratio, ultrasonic or microwave power, etc.) in the extraction with vegetable oils of phytochemicals from plant material is only one of the objectives related to the final oil extracts. The latter can be considered also as enriched or infused oils, as well as functional oils. The evaluation of their physical and chemical characteristics and indicators such as color, peroxide value (PV), para-anisidine and/or acid value (AV) is also important. It is worth noting that in many of the published studies of such oils, the antioxidant potential is assessed by indirect methods, such as DPPH, ABTS, FRAP and others. Such methods have no relationship with autoxidation but are claimed to provide information about antioxidant activity. The DPPH and ABTS assays measure a molecule’s capacity to act as a single-electron transfer agent or a hydrogen-atom donor, but significantly differ from the classical chain-breaking antioxidant mechanism. The latter requires an oxidizable substrate and the rate of autoxidation depends, among many other factors, on the concentration of oxygen dissolved in the lipid substrate. Much less research has been done on their oxidative stability, which should be sharply distinguished from the oxidative status assessed by PV, AV, or conjugated dienes and trienes amounts. On the one hand, these infused or enriched oils can be considered plant extracts, but on the other hand, one should not ignore the fact that lipid oxidation would be a problem that needs to be addressed.

Along with extraction conditions such as time, temperature, stirring speed (rpm), ultrasound or microwave power, solid-–iquid ratio, etc., which are usually the parameters for optimization, the nature of the solvent oil also has a quite determining and important role [21] (Table 1). Salazar-González et al. used bee pollen as a raw material and sunflower oil as green solvent for obtaining carotenoid-rich extracts [22] (Table 1). Varying the amount of pollen (from 5%wt. to 60%wt.) in the extraction mixture (24 h) led to results outlining the following trend: higher pollen concentrations allow for carotenoid richer extracts, but with significantly lower extraction yields. Pollen carotenoids are not near the saturation point in the sunflower oil, even at the highest proportion evaluated, but on the other hand, the decreasing trend of the extraction yield with respect to the matrix concentration is a typical behavior of diffusion-like extractions of compounds with limited solubility. In another study, pumpkin by-products were used as a source of carotenoids in which yield was assessed using three different oils (canola, corn and soybean oil) at different ratios from 1:10 to 3:10 g/mL oil [23] (Table 1). The trend showed a gradual decrease in carotenoids yield when the ratio increased. According to the authors, this may be due to the fact that vegetable oils became gradually saturated by a higher carotenoid concentration, occurring due to the mass transfer process reaching its maximum.

#### 3.1.2. Lycopene

Lycopene (Figure 1A) is a naturally occurring carotenoid found predominantly in tomato (*Solanum lycopersicum*) and tomato-based products, and possesses significant health-beneficial biological activities [24].

The classical approach to date for obtaining lycopene-enriched formulations was based on obtaining oleoresins by extraction with an organic solvent. After elimination of the solvent, although never completely, the extract obtained (diluted in oil or fat) could be used for further applications. To the best of our knowledge, WO Patent 2006/111591 was the first one related to a method for preparation of lycopene-enriched formulations free of organic solvents, based on the mixing of a lycopene source with lipid substrate. Using the patented method, Garrido et al. prepared lycopene-enriched olive and argan oils and studied their effect upon lipid serum parameters on Wistar rats, as well as the antioxidant status in humans after incorporation of the lycopene-enriched virgin olive oil into the diet [25,26] (Table 1).

The idea of using vegetable oils as green solvents for extracting lipophilic valuable substances can be considered from the different point of view, too, when the quality of these enriched oils has been improved as a result of the extraction. An example of that was the incorporation of industrial tomato waste (puree or tomato peel) in refined olive and sunflower oils [27] (Table 1). These enriched oils showed high thermal stability but a decrease in total phenols. In addition, some pro-oxidant activity was observed after incorporation of tomato puree. Data from a study published one year later revealed that tomato peels contained between seven and fifteen times more lycopene than tomato flesh (puree) and it could be suggested an effect of the lycopene content on oxidative stability of oil enriched by puree or tomato peel [28] (Table 1).

Grapeseed, peanut and extra virgin olive oils were also used and then compared for lycopene extraction from tomato waste, applying two methods: agitation and high-intensity ultrasound (20 kHz and 80% amplitude) [29] (Table 2). The authors concluded that the use of the ultrasound method was not desirable for the purpose due to formation of free radicals that oxidize the carotenoid. On the other hand, the olive oil allowed greater extraction ratios in comparison with grapeseed and peanut oils.

The solubility of lycopene and beta-carotene was reported by Borel et al. [30] to increase when the chain length of the triglyceride fatty acids decreased. Furthermore, no effect of the degree of unsaturation of the fatty acids on the extraction of carotenoids was observed.

#### 3.1.3. Astaxanthin

Due to its numerous biological functions including antioxidant and anti-inflammatory activities, astaxanthin has enormous potential for use in cosmetics, functional foods, and therapeutic medicine [31,32]. A biorefinery approach for its production from marine wastes, including step-wise application of fermentation, enzymatic digestion and solvent extraction, along with separation techniques, was recently reviewed by Routray et al. [33]. Aside from these methods, various vegetable oils (rice bran, coconut, groundnut, gingelly, mustard, soybean, palm and sunflower oil as well methyl esters of sunflower oil) were used as green solvents for the extraction of astaxanthin from shrimp processing waste [34,35,36] (Table 1). The aim of these experiments was limited to optimizing different extraction conditions and applying different models to achieve a higher degree of extraction of the target component. Crab processing waste was also used as a source of astaxanthin. Such extraction of snow crab (*Chionoecetes opilio*) discards was performed by Burke and Kerton [37] (Table 1) using corn, canola and sunflower oils, which gave between 24.85% and 37.93% astaxanthin recovery. In addition, the same authors used citric acid for demineralization of the material remaining after extraction, resulting in a pigmented protein powder as well as different proteases to deproteinize and isolate chitin. Besides crustaceans, algae and/or seaweeds are also considered an astaxanthin source [38,39,40] (Table 1).

#### 3.1.4. Crocetin and Crocins

**Figure 2:**
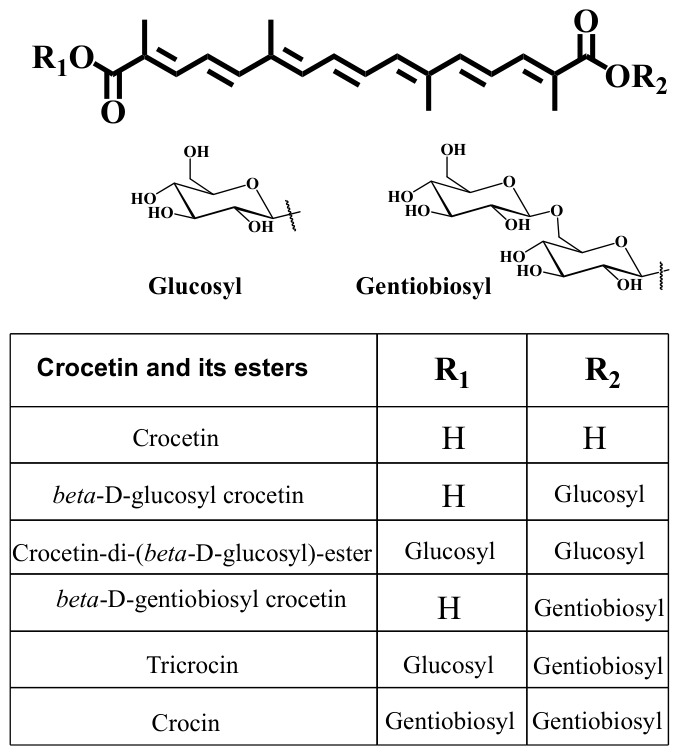
Chemical structure of crocetin and some of its ester derivatives.

Crocins, the glucosides of crocetin (Figure 2), are rare, plant-derived carotenoids isolated from saffron (*Crocus sativus* L.) and gardenia (*Gardenia jasminoides* L.) [41].

Crocins are amphiphilic compounds containing hydrophilic sugar moieties. The information about their extraction by plant oils is quite scarce. Research works focused on the development of water-in-oil emulsions or W/O nano-emulsions, incorporating *Crocus sativus* extracts [42,43] (Table 1). Regarding the extractant, water, extra virgin olive oil, and W/O (1:2 and 2:1 *v*/*v*) proportions were evaluated. Different distributions of the active ingredients, depending on the type of phase involved (water, oil, water/oil) and on the infusion time and temperature, were obtained. The oily phase was necessary to significantly improve the safranal enrichment at a temperature not higher than 80 °C. Crocins quantity was enhanced in 100% water and at low temperatures, while picrocrocin proved to be the most stable compound with extraction favored at high temperatures.

The publication of Slimani et al. [44] (Table 2) reports processing of *Crocus sativus* by-products subjected to ultrasonic extraction by sunflower oil. The effects of time, temperature and solid-solvent ratio on the phenolic compounds’ recovery were assessed by applying central composite design of RSM. The optimum extraction condition yielded the expected highest phenolic content (317.15 mg/kg), and antioxidant activity (89.34%).

### 3.2. Phenolic Compounds

Antioxidants can be classified according to various factors (origin, solubility, role in the organism) but the most important is the mechanism of their action, which in turn depends on their structures or, more specifically, on the functional group(s) responsible for the occurrence of antioxidant activity. Antioxidants acting as free radical scavengers are mainly aromatic compounds with relatively weak, i.e., labile–O–H, N–H as well as S–H-bonds. The majority of published information refers to phenols and polyphenols, and numerous studies have shown that the activity, in addition to their structure, depends also on their concentration, the properties of the oxidizable substrate and the oxidation conditions, including the oxidizing environment. Here, we consider some main classes of compounds possessing phenolic groups, a prerequisite for antioxidant activity, which are lipid soluble.

#### 3.2.1. Curcuminoids

**Figure 3:**
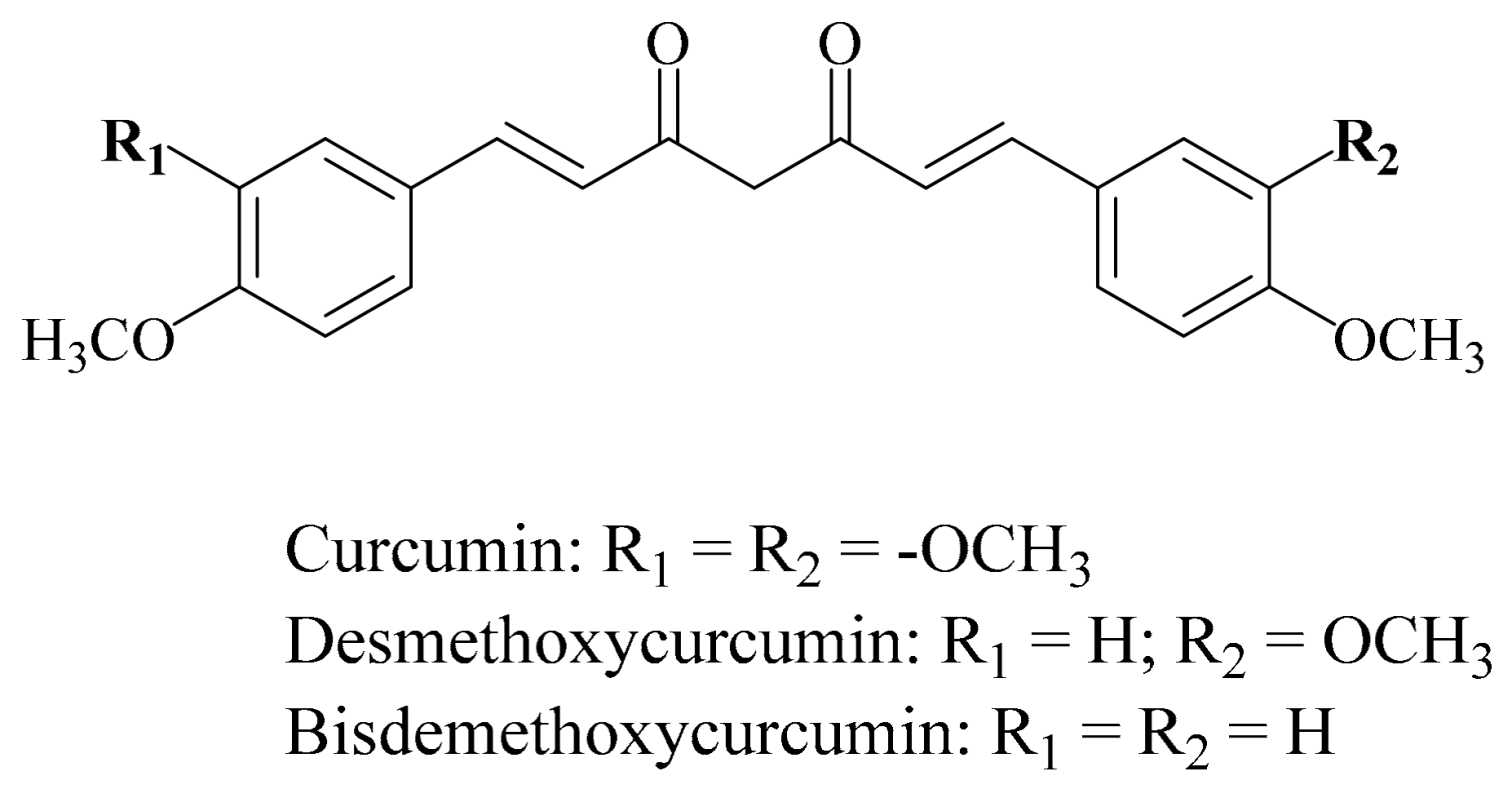
Curcuminoids found in turmeric (*Curcuma longa*) root.

Curcuminoids (Figure 3) are the main active components contained in the roots of the plant *Curcuma longa*, also known as turmeric, belonging to the ginger family (*Zingiberaceae*). The roots have been used for centuries as a source for the production of spices and colorings incorporated in many food products.

The research route on the antioxidant mechanism of action of curcumin was faced with the dilemma of whether the phenolic group(s) or the central methylene CH_2_-group of its heptadienone chain, are the first target for free radical attack. First, Jovanovic et al. [45] concluded that this CH_2_-group of the curcumin molecule is a H-atom donor both in acidic or neutral (pH ≤ 7) conditions and in acetonitrile. These conclusions were later refuted by Barclay and co-workers [46], who conducted comparative studies of curcumin and its structural analogues lacking phenolic groups, as well as three 2-methoxy-4-alkyl-phenols in chlorobenzene. Curcumin analogues without phenolic groups did not slow down the initiated oxidation of the substrates (styrene and methyl linoleate). A year later, in a subsequent publication by Jovanovic et al. [47], it was hypothesized that the radical, arising after the abstraction of the H-atom from the central active methylene group of curcumin, was resonancely stabilized to a phenoxyl radical. According to Litwinienko and Ingold [48], delocalization of the unpaired electron is implausible, since the structure of this radical is planar and should not allow intramolecular rearrangement accompanied by H-atom abstraction. In the same work, Litwinienko and Ingold introduced the concept for the role of sequential proton loss electron transfer, the so called SPLET mechanism, alternative to the known HAT, i.e., hydrogen atom transfer. These scientists also expressed the assumption that the contradictions regarding which was the active fragment of the curcumin molecule in reactions with free radicals arose as a consequence of different experimental conditions being used (especially reaction medium).

In laboratory experiments, soybean oil was enriched with 10% (*w*/*w*) of the following sources derived from turmeric and ginger: powders (commercial products), freeze-dried rhizomes and their peels [49] (Table 1). After centrifugation the supernatants were recovered and subjected to HPLC analysis, Folin–Ciocalteu assay and oxidative stability determination. Subsequently, the same amounts of oil in the same proportions to the exhausted (treated) powders and lyophilized samples were added in two consecutive cycles (cycle 2 and 3) under the same conditions as in the first extraction cycle (cycle 1). The maximum level of extractability of the bioactive lipid-soluble curcumin, 6-gingerol and 6-shogoal from the lyophilized samples was achieved during the first extraction cycle. Furthermore, the oxidative stability of soybean oils enriched with ginger and turmeric linearly increased with increasing phenolic yields and nonlinearly decreased with cycles.

Another study revealed that the temperature enhancement (from 60 °C to 70 °C) favored the enrichment of sunflower oil with curcuminoids, when dried turmeric rhizome was used as a plant source (solid to oil ratio 1:2), representing a percentage increase in curcuminoid content of 22.4% and ~26% for extraction time of 15 and 30 min, respectively [50] (Table 1). However, increasing twice the extraction time at both temperatures (60 °C and 70 °C) did not provide a higher degree of curcuminoid extraction. In addition, the authors performed comparative analysis of fatty acid composition and phenolic profile (by HPLC) of the non-enriched and enriched oils and determined their antiradical activity toward DPPH and ABTS radicals.

Mahmudah et al. [51] (Table 1) extracted phenols and carotenoids from turmeric powder (a commercial product) with extra virgin olive oil and virgin coconut oil varying dosage between 0 and 0.4 g/mL oil, and determined extractability of active compounds in both oils. The effects of temperature and heating time were studied and it was found that the higher the temperature and the longer the time, the higher the total phenols level, but the latter decreased after reaching an optimum point. Using these two oils, in a parallel study, the same authors applied ultrasonic extraction of turmeric with the addition of surfactant (Tween 80), at a dose of 0–30 μg per 10 mL oil and extraction time of 10–25 min [52] (Table 2). The results show that the vegetable oils with the addition of Tween 80 were effective for nano-emulsion formulations because they encapsulated curcumin. The maximum total phenolic content was achieved after 25-min extraction with 30 μg surfactant.

Microwave-assisted technique (at 800 W) was applied for the extraction of curcuminoids from dried organic *Curcuma longa* L. rhizomes using ten different oil samples [53] (Table 2). Among the oils, the most saturated one, coconut oil, was chosen for optimization of extraction conditions. These optimum conditions, giving maximal total curcuminoid content, were achieved at a ratio of 3:10 (gram per oil volume), in three irradiation cycles with a duration time of 1.5 min. The same conditions for curcuminoid extraction were investigated using almond, castor, peanut and rice bran oils, as well as with two types each of olive, sesame and sunflower oils. According to the results, castor oil extracted the highest content of bisdesmethoxy-curcumin, desmethoxy-curcumin, curcumin and total curcuminoid content, comparable to coconut oil.

The by-product left after curcuminoids extraction from turmeric oleoresin is known as the “*mother liquor*”. This curcuminoid-removed turmeric oleoresin (CRTO) consisted of residual curcuminoids and oil, and was used for enrichment and extension the shelf life of peanut butter [54] (Table 1). The oil, extracted from CRTO, contained volatiles as *ar*-turmerone, turmerone, and curlone, among other compounds, that likely act synergistically with the curcuminoids. The results indicated that such an approach is technologically promising as it provided an extension of the shelf life of the butter while maintaining its quality and safety, in addition to improving its nutritional content.

The utilization of waste and by-products from the agri-food industry aiming to extract valuable biologically active substances and therefore has remained in the spotlight over time, in parallel with the development of green technologies, including various new extraction techniques. Historically, unlike agricultural and food by-products, the medicinal and aromatic plants have been used for infusion, enrichment and/or flavoring of oils since ancient time [55]. However, science started to address this idea relatively late [56].

This review discusses different groups and classes of compounds that deserve special attention such as curcuminoids, cannabinoids and capsaicinoids.

#### 3.2.2. Cannabinoids

**Figure 4:**
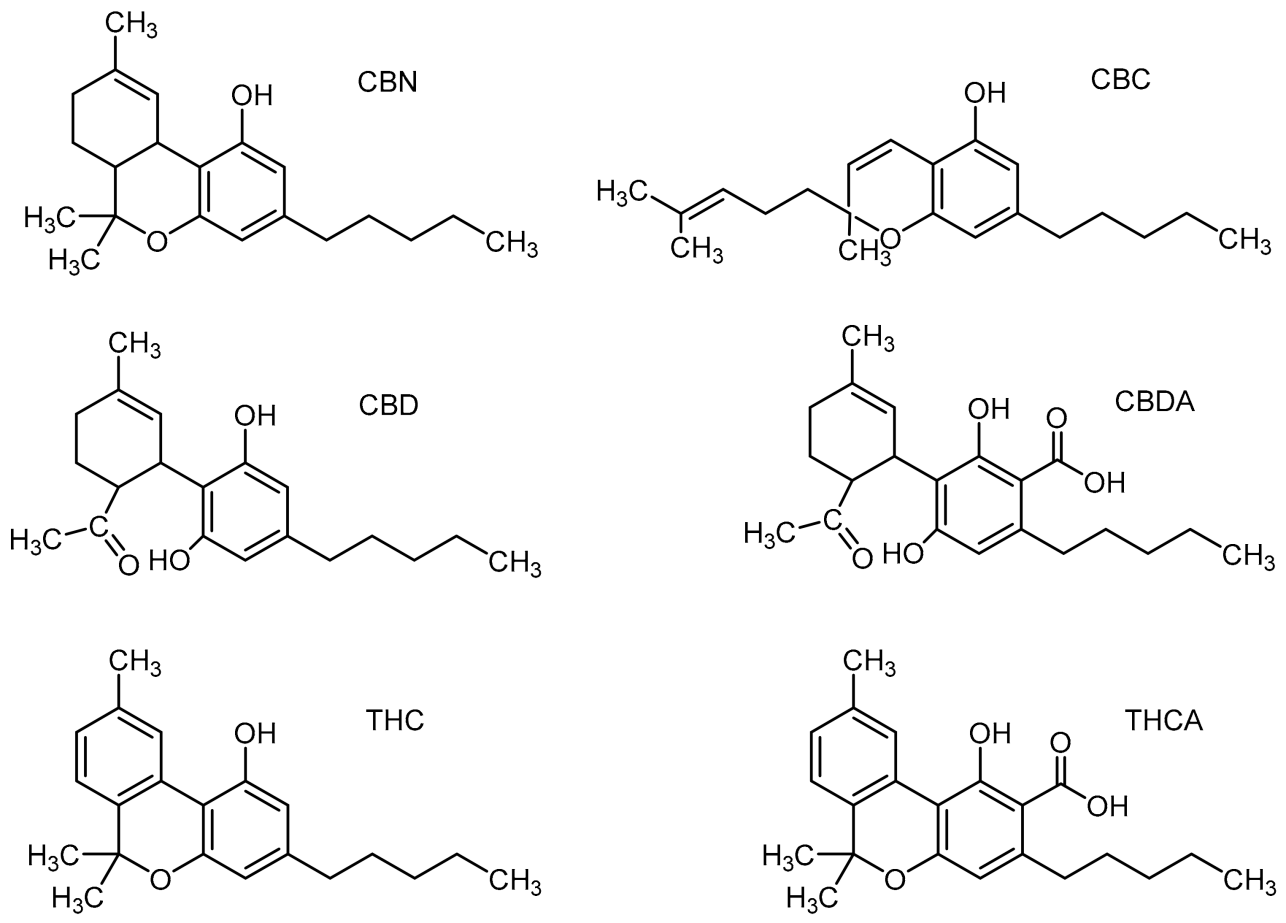
Chemical structures of cannabinoids found in *Cannabis sativa* L. Abbreviations: CBN—cannabinol, CBD—cannabidiol, THC—Δ^9^-tetrahydrocannabinol, CBC—cannabichromen, CBDA—cannabidiolic acid, THCA—Δ^9^-tetrahydrocannabinolic acid.

Cannabinoids are terpenophenolic compounds found primarily in the *Cannabis* plant (Figure 4). Although belonging to different structural classes they can be classified into two main categories: major and minor cannabinoids, which are present in cannabis in high and minor amounts, respectively. Cannabinoids exhibit distinct pharmacological effects, ranging from psychoactivity and pain relief to anti-inflammatory and neuroprotective properties [57].

Among the main cannabinoids discovered in *Cannabis sativa* L. are cannabinol (CBN), cannabidiol (CBD), Δ^9^-tetrahydrocannabinol (THC) and cannabichromen (CBC). The psychoactive THC and the non-psychotropic CBD are present in the plant flowering tops together with their carboxylated forms Δ^9^-tetrahydrocannabinolic acid (THCA) and cannabidiolic acid (CBDA), respectively (Figure 4). When cannabis is exposed to heat, oxygen and light, the two acidic compounds progressively decarboxylate to THC and CBD. The combination of these two has demonstrated a higher activity than THC alone in treating neuropathic pain in multiple sclerosis as well as peripheral neuropathic pain, rheumatoid arthritis, sleep disturbances and depression and intractable cancer pain [58].

Cannabinoids are compounds characterized by a highly lipophilic nature, and it is no surprise that all the endogenous ligands of cannabinoid receptors identified so far are derivatives of long chain fatty acids. N-Arachidonoylethanolamine, commonly known as anandamide (AEA), is an endogenous fatty acid neurotransmitter and some of its polyunsaturated congeners found in the mammalians brain could activate the two primary G-protein-coupled receptors CB1 and CB2 of the endocannabinoid system. Activation of the latter, CB2, is associated with anti-inflammatory and antioxidant effects [59].

Concentrated extracts of herbal cannabis became known as “Cannabis oil” because of its sticky and viscous appearance, obtained by solvent extraction of the buds or leaves of the cannabis plant. Examples include extractions with non-polar organic solvents like naphtha or petroleum ether and also with olive oil. Cannabis oils, often used for self-medication, are outside the scope of quality control and standardization. Romano and Hazekamp [60] (Table 1) drew attention to the fact that none of the methods for producing cannabis oil was validated in the published literature and no reports were made on the chemical composition of these products. The authors reported results on the effects of preparation methods, and in particular the solvents used (ethanol, naphtha, petroleum ether and olive oil) on the final composition of the cannabis oils obtained. Results revealed that most extracts contained 5–10% THC of total THCA + THC content with the exception of the naphtha extract. The latter was found to contain 33% of total THCA + THC content present in the form of THC. A possible explanation of this result was that the chemicals for stabilization, added to the naphtha, were responsible for the observed difference compared to petroleum ether. In addition, cannabis plant contains a wide variety of terpenes that may act synergistically with cannabinoids. On the other hand, extraction with olive oil was found to be the most beneficial based on the fact that it ensured higher amounts of terpenes than naphtha, petroleum ether, and ethanol [60]. Using European Pharmacopeia olive oil, Pacifici et al. [58] (Table 1) followed the procedure used by Romano and Hazekamp [60], and compared the oil with cannabis tea, obtained according to a protocol described in the Ministerial Decree of the Italian Ministry of Health (on medical cannabis). Furthermore, UHPLC-MS/MS analysis of cannabinoids content in cannabis tea and cannabis oil showed that extraction efficiency of oil was significantly higher than that of water with respect to the different cannabinoids. As a whole, their recovery from the tea samples was poor and variable, especially that of THC, with the ratio between the THC and CBD substantially different from that in the standardized flowering tops (THC/CBD of 0.25 in tea samples vs. THC/CBD of 0.71). In the oil preparations, the ratio THC/CBD was higher (0.49) than that of the tea sample (0.25) due to a higher THC recovery from herbal strains, and the stability of cannabinoids was acceptable during the first two weeks of storage (at 25 °C).

Extraction efficiency of the main bioactive compounds from *Cannabis sativa* when using various vegetable oils, namely the highly unsaturated omega-3 sacha inchi (*Plukenetia volubilis*) and perilla (*Perilla frutescens*) oils, as well as sesame seed oil, two types of olive oil, rice bran oil and coconut oil, was compared to that of organic solvents like ethanol, hexane, light (b.p. 40−60 °C) and heavy (b.p. 60−80 °C) petroleum ether [61] (Table 1). Vegetable oils are used in the development of the so-called Deja formula (ganja oil), which applies the method of heating and frying the flower of *C. sativa*. Chromatographic fingerprinting showed that the organic solvent extracts and vegetable oil-based cannabis samples had similar cannabinoid profile(s). High-performance thin-layer chromatography (HPTLC) analysis demonstrated that four bands were detected in all samples, indicating the presence of CBD, THC, CBN (Figure 4) and cannabigerol (CBG). Among the oil extracts, specifically those based on rice bran oil demonstrated the strongest radical scavenging activity against the DPPH stable radical [61]. The effect of the method of heating and frying (H and F method) decarboxylated cannabis powder in oils (30 min at 250 °C) was also investigated compared to conventional maceration in oil for 24 h. This H and F-assisted extraction significantly increased CBD and THC contents.

Analytical procedure based on the combination of headspace-solid-phase microextraction (HS-SPME) coupled to GC–MS and HPLC-HRMS (Orbitrap) was set up by Calvi et al. [62] (Table 2), validated and applied for the in-depth profiling and fingerprinting of cannabinoids and terpenes in two authorized medical grade varieties of *Cannabis sativa* L. (Bedrocan^®^ and Bediol^®^) and their macerated oils. Up to 100 volatile compounds composed their volatile fingerprints of which 72 were identified terpenes. The same authors summarized and reported the content of cannabinoids, terpenes and lipid oxidation products extracted from oil preparations based on extractions using three different methods, as well as their trend during 6-week storage (at 4 °C).

Bedrocan^®^ and Bediol^®^ medical cannabis varieties were used by Ramella et al. [63] (Table 1) for extraction with olive oil and MCT oil, and then were characterized by the different proportions of major cannabinoids (Bedrocan^®^: 22% Δ^9^-THC, <1% CBD, and Bediol^®^: 6.5% Δ^9^-THC, 8% CBD). During the first 60 days of storage (at 4 °C), THC and CBD were stable in both oils (olive, and MCT), regardless which of the two cannabis varieties was used for the extraction. An increase of CBN was registered after 45 days, due to the contemporary oxidation of THC. In addition to targeted cannabinoids, untargeted ones, terpenes and lipid degradation products were also analyzed using LC HRMS-Orbitrap and HS-SPME coupled to GC-MS. The terpenes extracted by using MCT from Bedrocan^®^ and Bediol^®^ were more abundant than in olive oil.

The possibility of using an ultrasonic homogenizer and sonotrode in extraction methods alternative to hot maceration was studied by Casiraghi et al. [64,65] (Table 2).

Following both innovative and existing extraction protocols, Ternelli et al. [66] (Table 1) aimed at chemical characterization of the obtained cannabis oils with a primary focus on cannabinoids and terpenes. Their study highlights the importance of the presence or absence of a decarboxylation step (temperature/time applied) as well as other sample pretreatment methods such as ethanol pre-extraction or steam distillation with subsequent removal of residual water. The latter was compared with a modern new microwave-assisted hydro-distillation technology. It is important to note that the distilled essential oil(s) obtained by both distillation methods were added to the final product (olive oil extract). An interesting approach was the one using ethanol pre-extraction by the following procedure: 80% of the inflorescences weight was submitted to a heating step in an oven in a sealed flask at 115 °C for 90 min. The remaining 20% of the inflorescences weight was extracted with ethanol, subsequently evaporated until a resin/paste was obtained, which was submitted to a heating step at 115 °C for 30 min (in a sealed flask). Then, olive oil was added to the resin/paste and to the inflorescences, and the resulting mixture was left to macerate under mechanical stirring for 24 h at room temperature [66].

Different pretreatment procedures of *C. sativa* inflorescences and extraction conditions led to a different oil product in terms of both cannabinoids and terpenes. In general, when the decarboxylation step was included in the extraction protocol, the yield of THC raised at the expense of the native acidic compound THCA, but harsh decarboxylation conditions resulted in an oil almost deprived of terpenes [66]. A direct maceration (at room temperature) of the inflorescences, previously decarboxylated at a moderate temperature in a closed system and partially pre-extracted with ethanol, provided the highest efficiency for both cannabinoids and terpenes extraction.

#### 3.2.3. Capsaicinoids

**Figure 5:**
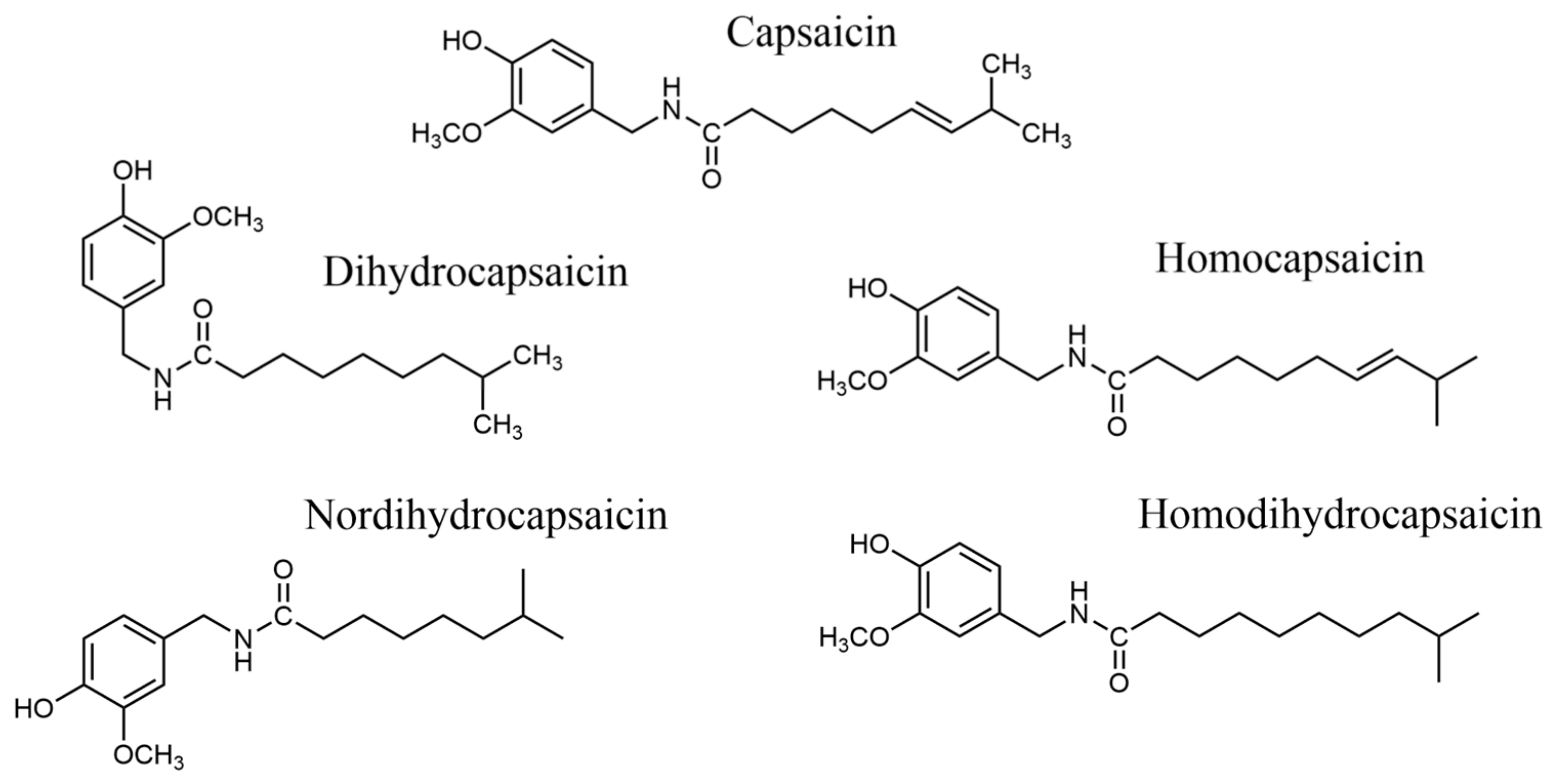
Chemical structures of capsaicinoids.

Capsaicinoids (Figure 5) are a group of lipophilic alkaloid compounds with a hot and spicy taste. A naturally occurring vanilloid, 8-methyl-N-vanillyl-*trans*-6-nonenamide, with the trivial name capsaicin, was initially isolated (in a crude form) from chili peppers (genus *Capsicum*). Capsaicin and dihydrocapsaicin comprise about 90% of the total capsaicinoid content in chili peppers (*Capsicum annuum* L.), while nordihydrocapsaicin, homocapsaicin and homodihydrocapsaicin are present in minor amounts. The fruits of *Capsicum annuum* have been used as a tonic, antiseptic and stimulating agent, to treat dyspepsia, appetites and flatulence, as well as to improve digestion and circulation due to the bioactive properties of capsaicinoids, including antioxidant, antimicrobial, antihyperglycemic and antihypertensive activities [67].

The main structure features responsible for the antioxidant activity of capsaicinoids are the *ortho*–methoxy–phenolic moiety, the benzylic C–H bond(s), and the chain C=C bonds (Figure 5), which can favor the radical adduct formation by direct addition of hydroperoxyl radical. The following positive antioxidant effects of capsaicin were observed in rats and guinea pigs fed a high-fat diet: suppression of lipid peroxidation and improvement the status of non-alcoholic fatty liver disease (NAFLD), decrease in the serum malondialdehyde (MDA) content and increase of superoxide dismutase, i.e., SOD activity in a dose-dependent manner. Other in vivo antioxidant mechanisms of action of capsaicin are related to blocking of low-density lipoprotein (LDL) peroxidation, prevention of glutathione (GSH) from oxidation by ROS and thus reducing the oxidative stress and enhancement/reinforcement of cellular antioxidant capacity.

The antioxidant effects of capsaicinoids were demonstrated in olive oil flavored by powder of dried red-hot chili pepper (*Capsicum annum*) [68] (Table 1). The tested samples were prepared as 10% and 20% infusions and were evaluated for 30 days. Results revealed that the antioxidant activity of the olive oil (determined by the ABTS^+∙^ radical method) greatly increased by the addition of chili pepper with a trend similar to that of capsaicin and dihydrocapsaicin concentrations changes during time. This green extraction process was intensified by applying ultrasound- or microwave-assisted procedures [69] (Table 2). Then capsaicinoids analysis of the oil aromatized with 20% chili pepper resulted in about 250 ppm capsaicin and dihydroxycapsaicin after 20 min treatment by ultrasound or 60 s by microwaves. Although the content of capsaicinoids extracted by traditional infusion (7 days of maceration) was higher (about 320 ppm), the ultrasound and microwaves processing seemed to be very effective, significantly more rapid and low-cost for the production of flavored olive oil.

In another study the olive oil was used for ultrasound-assisted extraction (UAE) of the pulp of hot pepper paste [70] (Table 2). The total capsaicinoids along with carotenoids and phenolic content were measured in respect to various extraction conditions including 8 h of maceration. Compared to the latter, UAE was found to produce higher amounts of extract in a shorter time and better antioxidant activity (measured by ABTS^+^ radicals inhibition). In a similar way, capsaicin, carotenoids and phenolic compounds were obtained by UAE from cumari-do-Pará peppers (*Capsicum chinense* Jacq.) using as green solvents the vegetable oils from soybeans, Brazilian nuts (*Bertholettia excelsa* H.B.) and palm olein, in comparison with extraction by common organic solvents (petroleum ether, acetone, ethanol and some their aqueous mixtures) [71] (Table 2). It was concluded that the vegetable oils were suitable for extracting and preserving bioactive pepper compounds, including the antioxidants capsaicin, carotenoids and phenolics. Among the tested green solvents soybean oil was the most effective one.

### 3.3. Oily Extracts of Medicinal/Aromatic Plants

A substantial part of the medicinal/aromatic plants are volatile aroma compounds (VACs). Usually, they include as main components various low-molecular-weight alcohols, aldehydes, esters, terpenes and phenols, the latter possessing strong antioxidant activity. VACs can easily migrate into vegetable oils thereby contributing positively to their functional properties, especially by increasing the total content of antioxidants. Instead of doing steam distillation prior to addition of essential oils into the basic (usually vegetable) oils, commonly known as carrier oils, aromatic plants could be directly put into them. For this purpose, various procedures have been developed and optimized, including maceration alone (Table 1) or combination with intensified techniques as stirring, ultra-sonication, etc. (Table 2). The oils used for extraction of such valuable components are most often olive oil [72,73,74,75,76,77,78,79,80] and sunflower oil [75,76,81], but peanut, rapeseed, grapeseed oils [75,76], corn oil [82], coconut oil [77], almond, hazelnut, apricot, avocado, soybean and wheat germ oils [75] have also been tested. Thus, as early as 1997 Antoun and Tsimidou [72] assessed oregano, rosemary and garlic-flavored (2% *w*/*w*) olive oils for their oxidative stability and consumer acceptance (Table 1). The authors found that the untreated oil reached a peroxide value (PV) of 70 meqO_2_/kg oil after 5 months of storage at 37 °C while the oregano and rosemary flavored oil samples had four- and nine-times lower PV, respectively. On the other hand, garlic extract neither increased nor decreased the olive oil oxidative stability. Four years later, Damechki, Sotiropoulou and Tsimidou [73] published results of their study on the presence of antioxidant and pro-oxidant substances in oregano and rosemary flavored (5% *w*/*w*) olive oils and found that their total polar phenolic content increased after infusion with the herbs by 3.5 and 1.7 times, respectively (Table 1). HPLC analysis of the methanol–water fraction of the enriched olive oil samples showed the absence of rosmarinic acid, but the presence of vanillic acid. A significant increase of pheophytin was found in the oregano-flavored oil, along with a higher carotenoids content (alpha-, beta-carotene and lutein), compared to the negligible increase observed in the rosemary one. The latter was more stable than the oregano oil under photooxidation conditions, which suggested a pro-oxidant role of the chlorophyll pigments such as pheophytin.

Yfanti et al. [74] also found that maceration of the aerial parts of oregano, rosemary and sage in olive oil increased its phenolic content by 1.3 to 3.4 times, improving significantly the free radical scavenging and antioxidant capacity of the infused oil (Table 1). The effects of temperature and heating time on the extraction were examined during maceration of moringa leaf (*Moringa oleifera* Lamk.) in extra virgin olive oil and in virgin coconut oil [77] (Table 1). The results revealed a successful extraction in the oil of flavonoids, phenolics, terpenoids, alkaloids, steroids and tannins, with the highest total phenolic content in the moringa leaf extract in virgin olive oil achieved at 50 °C and heating time of 2 h. The same temperature (50 °C), but for 30 min, was used during extraction with olive oil of 21 Algerian medicinal and aromatic plants (among them wormwood, palypade, mastic, laurel, thyme, mint, eucalyptus, verbena, lavender, sage, rosemary leaves, etc.) in order to evaluate their reducing power measured as the radical scavenging activity toward DPPH-radical [78] (Table 1). The results revealed that the oil infusion of seven plants (case of *Artemisia absinthium*, *Polypedium vulgare*, *Pistacia-lentiscus*, *Laurus nobilis*, *Thymus algeriensis*, *Thymus vulgaris* and *Mentha spicata*) possessed an antiradical activity higher than that of the olive oil.

In a similar study but applying an ultrasound-assisted maceration the olive oil was aromatized with freshly cut basil leaves resulting in reduced time from hours or even days to few minutes, with similar GC/MS profiles of the compounds in the macerated and ultrasound-assisted macerated oils [79] (Table 2). Olive oil was enriched with phenolic acids, mainly rosmarinic, o-cumaric and vanillic, also by immersion of dried oregano leaves using optimized procedures by magnetic stirring and sonication thus improving the antioxidants extraction from the plant [80] (Table 2).

Evaluation of the dissolving power of ten vegetable oils: rapeseed, peanut, coconut, grape seed, olive, jojoba, avocado, sunflower and olive oils for extraction of volatile aroma compounds from dried sweet basil leaves (*Ocimum basilicum* L.) was assessed by Li et al. [76] (Table 1). The main components of VACs were linalool, eugenol, estragole, eucalyptol, limonene and trans-anethole. Since vegetable oils were considered as alternatives to dichloromethane for extraction of VACs, the predictive Hansen solubility parameter (HSP) of all solvents and solutes were compared for a fast-theoretical selection of the oils, and the sunflower oil was theoretically and experimentally proven as a preferential solvent in this study. Some years later the experimental results on green extraction of volatile and non-volatile compounds from rosemary leaves were published, too [75] (Table 1), revealing that among twelve refined vegetable oils the soybean oil ensured the highest total phenolic content (TPC). Carnosol, carnosic and rosmarinic acids were the main antioxidants and their extractability was investigated similarly using the soybean oil containing soy lecithin or glyceryl monostearate/monooleate as additives, which could enhance the oleo-extraction of these non-volatile antioxidants by 66.7%. The additives helped also to improve the solvation of VACs (e.g., borneol, camphor, o-cymene, eucalyptol, limonene, *alpha*-pinene and terpinen-4-ol) by 16%.

Rosemary (*Rosmarinus officinalis*), garden sage (*Salvia officinalis*), summer savory (*Satureja hortensis*), laurel (*Laurus nobilis*) and other aromatic plants were put in olive oil and exposed to ultrasounds for their enrichment with valuable plant components. The content of extracted polyphenols, chlorophylls and carotenoids was spectrophotometrically measured [83].

## 4. Achievements and Challenges

Vegetable oils are increasingly being used as a preferred alternative to organic solvents for the extraction of biologically active compounds, e.g., antioxidants, in the food, pharmaceutical and cosmetic industries due to their undeniable advantages:

✓ They are sustainable, green solvents characterized by biodegradability, biocompatibility and safety, with excellent green metrics. For example, Vinas-Ospino et al. [18] compared different green solvents, including vegetable oils (sunflower and olive oil), fatty acids and deep eutectic solvents (DES) with hexane as a conventional organic solvent for carotenoid extraction from orange by-products. Green metrics were used to evaluate, among many factors, the environmental impact, safety and cost of the solvents used according to the Eco-Scale tool proposed by Van Aken et al. [84] and the Blue Applicability Green Index (BAGI), proposed by Manousi et al. [85]. The results revealed that all the green solvents scored over 75 of a total of 100, demonstrating exceptional reaction conditions. According to the cost/efficiency parameter, vegetable oils are most affordable option, at that without the need of pre-treatment.
✓ Plant oils have a strong affinity for lipophilic compounds. This extraction selectivity represents a major advantage, allowing for the production of extracts enriched with target non-polar compounds.
✓ The application of vegetable oils for extraction of lipid soluble phytochemicals from bioresources results in ready-to-use extracts, thus eliminating the need of energy consuming steps after solid-liquid separation like solvent evaporation from both, from the final extract and spent plant residue, and solvent purification.
✓ According to the cost/efficiency parameter, vegetable oils are the most affordable option and require no pre-treatment. Furthermore, while the preparation of green solvents such as DES, natural deep eutectic solvents (NADES) and ionic liquids requires availability of precursors and laboratory treatment, edible oils are directly accessible at commercial market prices.

Along with these advantages, vegetable oils also have some limitations as extraction media:

○ Compared to the traditional organic solvents like hexane, vegetable oils possess higher viscosity, which generally complicates the extraction process because of mass-transfer limitations and reduced extraction kinetics. This challenge can typically be overcome by the application of auxiliary technologies, including ultrasound- and microwave-assisted extraction procedures, precisely controlling the amount of moisture.
○ Oil recovery presents another difficulty after extraction. In this context, the impact of the solid-to-liquid ratio is inversed: a higher ratio increases antioxidant extraction efficiency but simultaneously leads to greater oil adsorption onto the biomass.
○ When certain inherent sensory properties of extraction oil, such as flavor and color, are undesired in the final product, additional treatment or selection of an alternative oil is required.
○ A significant challenge during extraction with vegetable oils is their oxidative stability, which strongly depends on their content of unsaturated fatty acids. The latter are prone to autoxidation, leading to the deterioration of their properties and overall quality. This is directly related to the shelf life of the oils and their extracts.

Applying edible vegetable oils for the extraction of lipid soluble phytochemicals from bioresources could result in ready-to-use extracts whose valuable physical, chemical and/or biological properties determine their suitability for inclusion in the composition of a final product. Until now, the production control of such oils has been based on established ranges for key chemical indices such as peroxide value, acid value, iodine value, para-anizidine value and so on. These parameters are a measure of the initial oxidation degree and the oxidative status of a given vegetable oil, but must be sharply distinguished from their oxidative stability. The stability of lipids is expressed as the storage time at certain conditions before the first signs of rancidity appear and requires a kinetic method for testing. Enrichment of oils by adding antioxidants when directly extracted from natural sources can lead to both the increase and the decrease in oxidative stability. For example, carotenoids may lose their antioxidant effectiveness at high concentrations and act even as pro-oxidants at high partial pressure of oxygen. Furthermore, in addition to the extraction of the target compounds from natural sources, other concomitant components are also extracted. Their possible synergistic or antagonistic effects should also be anticipated. Most of the published scientific data on the problem concern the selection of specific target compounds (carotenoids, curcuminoids, capsaicinoids and others), but during an oil-based extraction process, in addition to the target molecules, many other lipid-soluble substances are extracted, the influence of which may have a catalytic effect on the oxidation process. Thus, the overall antioxidant capacity of enriched and infused oils is a result of the extracted phytochemicals on the one hand, and the intrinsic (native) antioxidants of the vegetable oils themselves on the other. Most published scientific studies compare oils before and after extraction. Untreated oils are used as control samples; however, to assess the role of their intrinsic antioxidants, the oils should be stripped (e.g., by applying adsorption column chromatography) and used as a blank sample.

**Figure 6:**
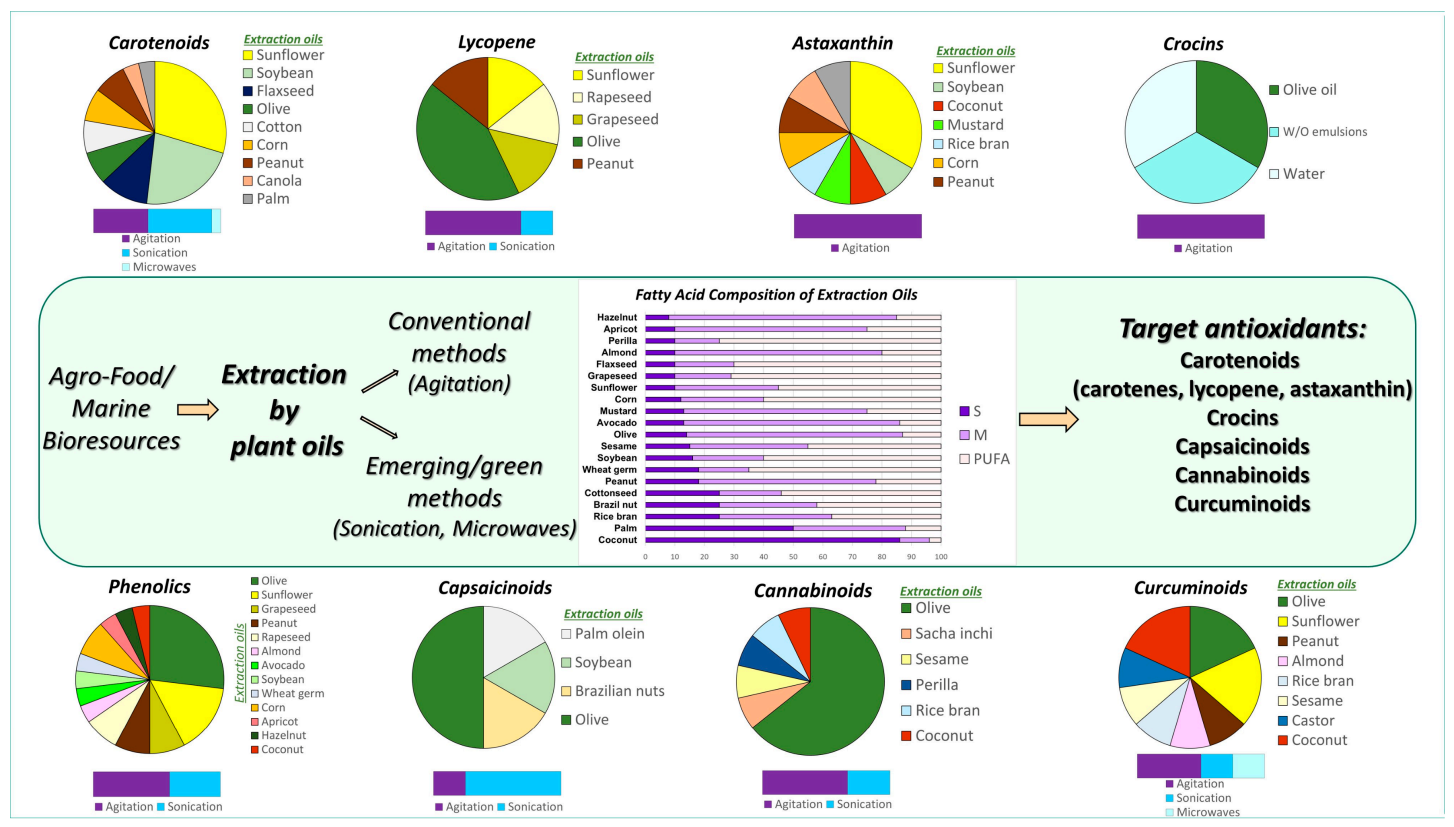
Summary of the data in Table 1 and Table 2 presenting which oils and which extraction techniques have been used for extraction of each antioxidant, and how often.

An overview of the references discussed in this review indicates that olive and sunflower oils have been the most widely used extraction media, appearing in 27 and 18 studies, respectively. Figure 6 graphically summarizes this information (included also in Table 1 and Table 2) and clearly shows which oils, as well as which extraction techniques, have been most frequently applied for the extraction of each antioxidant.

Analysis of these data reveals that, firstly, more than half of the extractions have been performed using a single plant oil, thereby making a comparison between results is impossible; and secondly, in studies where several oils have been used and compared, the conclusions and recommendations about the application of a given oil for the optimal extraction of antioxidants from agro-food and marine bioresources are rather dispersed, and sometimes contradictory. For example, olive and soybean oils have ensured similar extraction yields of carotenoids from carrot, amounting to 63 and 64.4 mcg retinol equiv./L, respectively [12]. On the other hand, canola oil has extracted 62% of carotenoids from pumpkin, which represents a higher yield than those obtained with soybean and corn oils [23]. Carotenoids recovery from shrimp waste by five different oils was 26.3%, 24.8%, 24.3%, 23.1% and 16.1%, respectively, by sunflower, soybean, rice bran, peanut and mustard oils [36], whereas the mango pulp was better extracted by flaxseed oil compared to sunflower and peanut oils [17]. Similarly, the peanut oil was not recommended for extraction of lycopene from tomato biomass where olive oil gave better results [29]. Astaxanthin possessed extraction recovery from snow crab of 37.9% by corn oil, 31.2% by canola oil and 24.9% by sunflower oil [37]. Soybean oil was the most effective in extracting of bioactive pepper compounds, particularly carotenoids (35 mcg/g, 69% recovery) and capsaicin (39 mcg/g), respectively, followed by Brazilian nuts (24 mcg/g and 35 mcg/g) and Palm olein (22 mcg/g and 27 mcg/g) [71]. Curcuminoids were extracted from *Curcuma longa* by almond, castor, peanut, rice bran, olive, sesame and sunflower oils, with a recovery from 25.5% (sunflower oil) to 38.1% (castor oil) [53]. The effect of six vegetable oils as solvents on the cannabinoids (THC and CBD) extraction from *C. sativa* has been determined by Zen et al. [61]. The sacha inchi-enriched oil exhibited the highest content of THC (0.034% *w*/*v*) and CBD (0.017% *w*/*v*), followed by the coconut oil (THC 0.033% *w*/*v* and CBD 0.015% *w*/*v*). The sesame and perilla seed oils showed a significantly lower content of these compounds than the virgin coconut oil [61].

The diversity of results indicates that each extraction procedure should be optimized regarding the target antioxidant, the plant oil, the applied technique, the intended purpose of the final product, etc.

**Table 1:** Application of edible oils for extraction of phytochemicals from natural (re)sources using conventional techniques including maceration, infusion, agitation, etc.

Target Compounds	Natural Sources	Processes			Analysis	Ref.
		Sample Preparation	Oil Type	Extraction Conditions		
Carotenoids (lutein, *alpha*- and *beta*-carotene)	Carrots (*Daucus carota* L.)	Carrots (2 mm thick slices and 30–40 mm in diameter) were dried at 60 °C with an air velocity (1 m/s) for 22 h and ground (0.8 mm sieve)	Extra virgin olive oil and refined (100%) soybean oil	Extraction from the dehydrated matrix with edible oils were performed by agitation (at 10,000 rpm) in a blender at room temperature, followed by vacuum filtration in a Buchner funnel (150 mm diameter).	Total carotenoids determined by spectrophotometry and HPLC analysis of carotenoid profile; extraction efficiency and oil recovery; shelf life of enriched and not enriched oils; experimental design and statistical analysis	[12]
Carotenoids (lutein, *alpha*- and *beta*-carotene, *beta*-cryptoxanthin, 13-*cis-beta*-carotene)	Pumpkin pulp (*C. argyrosperma*)	The pulp, after discarding the seeds and peel, were cut into small pieces (about 2 mm thick); the slices were dried at 60 °C until moisture content below 10%, then milled and sieved (0.425 mm sieve)	Canola oil, corn and soybean oil	Dried pulp was mixed with oil at different ratios (1:10, 2:10, 3:10) and macerated for 90 min at 35 °C in the dark, under agitation (225 rpm); the mixture was then centrifuged at 3070× *g* for 10 min and filtered. through Whatman No. 1 filter paper.	Carotenoid extraction yield determination, and optimization of the extraction conditions	[23]
Carotenoids and chlorophylls (pigments)	Avocado (*Persea americana*) leaves from *Hass* and *Drymifolia* varieties	After sanitization, the leaves were lyophilized ground and sieved through 50 mesh sieves (0.297 mm)	Corn and sunflower oil	Oils were mixed with the avocado leaf powders at a 1:100 (*w*/*w*) ratio; extraction was carried out by mechanical agitation at 700 rpm for 6 h (room temperature); then extracts were filtered through Whatman filter No. 1 and centrifuged at 3700 rpm for 15 min at 10 °C.	Emulsification, micro-encapsulation’s preparation, physical and chemical properties characterization, and pigment quantification and anti-radical (toward ABTS•^+^) and FRAP activity assay	[68]
Carotenoids	Goji berry (*Lycium barbarum*)	Berries, preliminary soaked in deionized water, were crushed by a kitchen blender for 2 min, dispersed with a high-speed homogenizer at 10000 rpm for 3 min and finally the obtained puree was quickly frozen with liquid nitrogen at −20 °C for 2 h	Soybean, sunflower, palm, and cottonseed oil; medium chain triglycerides (MCT)	The frozen puree, taken out at 40 °C for 30 min to thaw, was mixed with a vegetable oil (at a ratio 1:2), stirred magnetically for 5 min at 25 °C and homogenized at 10,000 rpm for 3 min, subjected to freezing again (at −20 °C for 2 h), followed by thawing (40 °C for 30 min), and centrifugation (11,290× *g*, at 4 °C for 20 min); this extraction including freeze–thaw cycle(s) and centrifugation were repeated twice, and finally the supernatants were combined.	Rheological properties, fatty acid composition, differential scanning calorimetry (DSC) analysis to compare the thermal properties of enriched and non-enriched oils; light and thermal stability of carotenoid-enriched oils and measurement of carotenoid retention ratio	[21]
Total carotenoids	Bee pollen	The bee pollen was subjected to a drying and cleaning process.	Commercial sunflower oil	Extraction was carried by continuous stirring (at 500 rpm) of mixtures of the sunflower oil and bee pollen between 5 and 60%wt (g pollen/100 g mixture) during 24 h at room temperature in the dark; Three ratios with the highest carotenoid content (40%wt, 50%wt and 60%wt) were selected for a second set during which extraction was performed and monitored within 16 days for a kinetic study.	Carotenoids were determined spectrophotometrically; The effect of the pollen: oil ratio was evaluated, as well as the extraction kinetics	[22]
Carotenoids	Mango pulp (*Mangifera indica*)	Blanching at 75 °C and addition of pectinase and cellulase followed by enzyme inactivation and cooling ≤ 10 °C	Flaxseed, groundnut and sunflower oil	Mixing the enzyme-treated pulp with oils at ratios 1:2; 1:3; 1:4 at 27 °C applying magnetic stirring (1000 ± 10 rpm) for different periods: 30, 60, 90 and 120 min.	Antiradical activity (ABTS, DPPH, and FRAP assay) color values	[17]
Lycopene	Tomato pulp	Dehydration of the pulp	Virgin olive oil	The olive oil was mixed with the pulp in a weight proportion of 1:1 in a discontinuous industrial mixer (2500–5000 rpm), at different temperatures: 40°, 50°, 60°, 70°, 80° and 90 °C.	Rate of extraction; evaluation of the effect of lycopene-enriched virgin olive oil consumption on urinary antioxidant capacity in young, middle-aged and elderly participants	[25]
Lycopene	Tomato puree and/or tomato peel	The skin was frozen immediately after separating from the tomatoes’ flesh and the latter was discarded. Puree was purchased from the local market and all samples were freeze-dried	Extra virgin olive oil, refined olive oil and sunflower oil	Oil samples (20 g) containing 5%, 10%, 20% and 30% tomato puree were homogenized for 3 min and then centrifuged at 6240× *g* for 15 min; the oily phase was removed and stored at minus 20° until analysis. The peel was mixed with the refined olive oil only at 5%, and 10% and left to diffuse for 24 h at 4° in the dark and then centrifuged at 6240× *g* for 15 min.	The resulting enriched oils were analyzed for their oxidative status, determining by the peroxide value, the oxidative stability assessed by Rancimat test, antiradical activity toward DPPH radical and carotenoid analysis	[27]
Lycopene and other carotenoids	Fresh tomatoes hybrid *Raissa F1* (grown in greenhouses), oleoresin “Maxopene 10%” and processing waste (peels)	Fresh tomatoes were blended in a blender for 5 min.	Refined rape oil	Oleoresin and oil were mixed 1:800 with a magnetic stirred for 7 h at 20°. The tomato puree (paste) obtained after blending was mixed with oil 1:1 with a blade mixer for 2 h at 20°.	The solubility of lycopene contained in fresh tomatoes (*Raissa F1*) and oleoresin from tomatoes containing 10% of lycopene was compared	[28]
Astaxanthin	Snow carb processing discards (*Chionoecetes opilio*)	Crab processing discards, collected from a processing plant, were prepared and subjected to characterization by proximate analysis (moisture content, crude protein, crude lipid and ash)	Canola, corn, sunflower oil	An amount, about 100 g, of Crab by-product was blended with 50 mL distilled water and particles with a size of 1–5 mm were obtained. The blended samples were then mixed with oils at a ratio 1:1 (*w*/*v*) in jars and incubated for 2 h at 60 °C with continuous agitation (at 165 rpm). After incubation the samples were centrifuged for 10 min at 8000 rpm, at 20 °C. Finally, pigmented oil layers were decanted and filtered through Whatman 40 ashless filter paper.	Total astaxanthin content, Tristimulus color parameters	[37]
Carotenoid pigments (fucoxanthin, lutein, etc.)	Brown seaweeds (*Sargassum horneri* and *Saccharina japonica*), spinach (*Spinacia oleracea* L.) and olive leaf powders	After washing under running tap water, one portion was cut into small pieces. dried (24 h) at 60 °C in the dark and dried into powder which then was classified with a sieve in two particle sizes, 150–250 μm and <150 μm; another portion kept in boiling distilled water for 8 min and/or subjected to the following four-fold treatment: 15 min soaking of the seaweed sample(s) in 0.2% (*w*/*v*) citric acid solution, and after washing, soaking again, but in 0.2% (*w*/*v*) Na_2_CO_3_	High oleic sunflower oil, corn, olive, sesame, soybean, rice bran, rice germ, linseed, rapeseed oil, as well fish oil; Tricaprylin, tributyltin, and tricapronin	A total of 0.5 g of sample powder (<150 μm) was mixed with 1.0 g edible oil, vortexed for 1 min, and left (at constant temperature) in the dark. After a certain period of time, an oil sample was centrifuged for the separation of the oil from the residue.	Carotenoid analysis; extraction kinetics (effect of extraction temperature and time) and optimization of the extraction conditions, effect of seaweeds pre-treatment; effect of addition of an egg lecithin	[38]
Astaxanthin and its esters	Northern shrimp (*Pandalus borealis*) processing waste	Freeze drying FD4 process, homogenization (laboratory grinder) and then separated by vibrating sieves (0.02 mm < id < 10 mm).	Sunflower oil and methyl ester of sunflower oil	Extractions were performed by adding sunflower oil or methyl esters of sunflower oil in three different ratios to shrimp waste (10 g): 1:3, 1:6 and 1:9 at temperatures of 25, 45 and 70 °C. Samples were mixed with a mechanical stirrer (200 rpm) for 24 h. In a second set, the extractions were carried out with a fixed extraction time (3 h) and temperature 70 °C, and also at a constant ratio 1:9.	Quantification by HPLC and by spectrophotometric analysis; optimization of the extraction conditions (temperature, duration and ratio) in the first set; in the second set of experiments, using another batch (shrimp waste), the effect of particle size and moisture content as well as stirrer speed were investigated	[34]
Astaxanthin	*Haematococcus pluvialis* culture	*H. pluvialis* grown; After reaching a stationary stage, due to nitrogen deficiency, it was moved to a high-intensity photo-incubator to transform the green cells to red cysts	Soybean, corn, grapeseed and olive oil	A ten-days-incubated cyst (30 mL) culture was mixed with oil (30 mL). After vigorous stirring, the mixture was allowed to settle under gravity (at room temperature) and the oil extract, after decantation, was separated.	Quantification of astaxanthin	[39]
Astaxanthin	Giant tiger shrimp (*Penaeus monodon*) waste	The frozen shrimp waste was washed repeatedly in warm water, then freeze-dried, ground and sieved using set of sieves (40/60, 60/80, and 80/100 mesh)	Palm oil	A total of 300 mL of palm oil was introduced in three neck flask and heated in a temperature-controlled heating jacket at different temperatures: 50, 60 and 70 °C. After the desired temperature was reached, 50 g of shrimp waste at various particle sizes was added to the palm oil.	Extraction kinetics and thermodynamic parameters determination; the concentration of total carotenoid, presented as astaxanthin, in palm oil was measured spectrophotometrically	[35]
Astaxanthin	Shrimp (*Penaeus indicus*) waste comprising of head and carapace	The material was thawed in running water before use and homogenized in a laboratory mixer	Refined sunflower, groundnut, soybean, gingelly oil, mustard, coconut and rice bran oil	A total of 10 g of homogenized waste was mixed with 20 mL of oil and heated in a water bath at 70° for 2 h, the filtered and the filtrate was centrifuged at 3000× *g* for 10 min. The pigmented oil layer from the supernatant was separated.	Spectrophotometrically determination of astaxanthin in the range from 486 to 504 nm (depending on the oil); optimization of extraction conditions	[36]
Astaxanthin	Bulgarian green microalga strain *Coelastrella* sp., isolated from a stagnant water in metal trough near Varvara village, Bulgaria	The microalga was cultured and maintained as non-axenic monoculture	Sunflower oil	The algal suspension (from stationary phase 10th day) was mixed with the oil in 1:1 ratio for 48 h under continuous stirring (at room temperature). The mixture was allowed to settle under gravity into a separating funnel. The top layer, after separation, was centrifuged at 4000 rpm at 10 min.	Anticancer and apoptogenic activity in vitro against human tumor cells HeLa by means of MTT and fluorescence microscopy analyses	[40]
Crocins (trans-crocin and picrocrocin), safranal and flavonols (kaempferol, quercetin and their glucosides)	Saffron species (*Crocus sativus* L.) flowers	Saffron was ground	Extra virgin olive oil	A total of 0.25 g of ground saffron was added to 15 mL oil (extractant); the extraction was carried out in glass tubes using an incubator allowing efficient temperature and vortex control; four extractant media were used: water, oil, and water/oil in 1:2 and 2:1 (*v*/*v*) proportions (at 60, 80, and 100 °C).	LC-QTOF MS/MS analysis (liquid chromatography-quadrupole time-of-flight tandem mass spectrometry) of the extracts and crocins’ profiling	[42]
Crocins (trans-crocin and picrocrocin), flavonoids (kaempferol 3-O-sophoroside)	Saffron (*Crocus sativus* L.)	Saffron was ground and homogenized	Extra virgin olive oil, water, water/oil	A total of 25 g saffron was extracted with 25 mL oil, water or water/oil mixtures, at various condition according to temperature (60 °C, 80 °C, 100 °C), infusion time (10 to 30 min) and the composition of the medium (water, oil and water/oil).	LC-QTOF MS/MS methods were applied	[43]
Phenolic compounds, including gingerols, shogaols and curcuminoids	Ginger and turmeric spices (commercial products), and fresh rhizomes of ginger and turmeric	The rhizomes were manually peeled, cut into small pieces, freeze dried at minus 40 °C and grounded (the peels were also cut, freeze dried, grounded)	Refined soybean oil	Ginger and turmeric powders were mixed with the oil at concentration 10% (*w*/*w*), for 10 min, using disperser tool, and centrifuged for 10 min at 5000 rpm at 4 °C. The supernatants consisting of soybean oils supplemented with powders were recovered (Cycle 1) while the exhausted powders were recycled two times by adding soybean oil and following the same enrichment (Cycles 2 and 3).	Total phenolic content, HPLC analysis, antiradical activity toward the model radical DPPH and oxidative stability	[49]
Phenolic compounds	Turmeric powder (*Curcuma longa* L.), commercial product		Virgin coconut oil and extra virgin olive oil	Turmeric samples with a dosage variation of 0–40% (gr/mL) were added into a 100 mL measuring flask, mixed with the oil (to the limit mark), stirred, and heated for 2 h at 50 °C. Next, the solution was strained through a cheesecloth.	Total carotenoid and total phenolic content	[51]
Curcuminoids	Turmeric rhizome (*Curcuma longa* L.)	Sunflower seeds were crushed to obtain particles with an average diameter of 0.60 mm	Sunflower seed oil	After preliminary preparation of sunflower oil (blank) from the seeds using ethyl acetate as a solvent (in a ratio of 1:8 g/mL) at 60 °C for 1 h, two extraction strategies were applied: extraction of the compounds from the plant material using the obtained sunflower oil at 1:2 (g/g) at different temperatures (60 °C and 70 °C) and time (15 and 30 min), and simultaneous extraction of sunflower seed oil and turmeric rhizome using ethyl acetate as solvent at a ratio 1:4.5:8 (g/g/mL), respectively (at the same temperatures and extraction time periods).	Spectrophotometric determination of curcuminoid content and total phenolic content, study of antioxidant potential and phenolic profile with UHPLC-MS/MS analysis (ultra- high-performance liquid chromatography coupled to a triple-quadrupole mass spectrometer)	[50]
Curcuminoids and others (aromatic oil, extracted from CRTO, containing turmerone, and curlone)	Mother liquor, after industrial isolation of curcuminoids and referred as curcumin removed turmeric oleoresin CRTO, consisted of residual curcuminoids and oil	Peanuts, after roasting for 20 min at 120 °C, were dehulled and ground (sugar and salt were added at 5% and 1% by weight)	Peanut butter	Preparation of enriched peanut butters was achieved using a range of concentrations for CRTO oil and CRTO (from 0.15% to 0.35%), and curcuminoids (from 0.045% to 0.2%); the optimal concentrations, based on their impact on bitterness and color, were determined to be 0.25% for CRTO and CRTO oil, and 0.045% for curcuminoids.	Proximate analysis, color measurement, antioxidant activity and microbial analysis including detection of yeasts and molds detection and coliform analysis	[54]
Cannabinoids, monoterpenes and sesquiterpenes	*Cannabis sativa* (medicinal cannabis), “Bedrocan” variety, female flower tops were used, i.e., *Cannabis* Flos	Dried flowers were manicured to remove leaves and stems, cut into smaller pieces, and finally homogenized by grinding; without application of a preheating step	Olive oil and water/olive oil 70:20 (*v*/*v*)	*First extraction*: 5 g of plant material in 20 mL of oil and 50 mL of water heated in a water bath at ~98 °C for 60 min.; allowed to cool before filtering; during the filtering (by pressing) the plant material was rinsed with 20 mL of hot water. The mixture was placed in a freezer (−20 °C) overnight to separate the water from oil phase. *Second extraction*: 10 g plant material in 100 mL oil were heated in a water bath at ~98 °C for 120 min and allowed to cool before filtering.	GC-FID determination of terpenes, and HPLC analysis of cannabinoid profiles	[60]
Cannabinoids and terpenes	*Cannabis sativa*, “Bedrocan” and “Bediol” medical varieties	Inflorescence grinding and decarboxylation at 125 °C for 30 min. in an oven	Olive oil and MCT oil (medium chain triacyl-glycerol)	A total of 5 g of both medical *Cannabis* varieties were added to the oil in a 1:10 ratio (plant/oil), the mixture was shacked for 10 min (at 25 °C and 60% RH) using mechanical rod stirrer, and finally extracted at 100 °C for 30 min.	HPLC-Q-Exactive-Orbitrap-MS analysis of cannabinoid and HS-SPME and GC-MS terpene analysis	[63]
Cannabinoids	*Cannabis sativa*	*C. sativa* was ground into powder and subsequently decarboxylated at 110 °C for 60 min	Sacha inchi, virgin coconut, sesame seed, perilla seed, rice bran oil, and olive oils of high-heat cooking, of roasting and frying grade	A total of 5 g of decarboxylated *Cannabis* powder in a ratio 1:10 with the oil, macerated for 24 h; Simultaneously, another 5 g of the decarboxylated cannabis powder was fried in vegetable oils for 30 min. at 250 °C. Then, all the fried powder materials were re-soaked for 24 h in the respective vegetable oils and the supernatant was filtered.	Cannabinoid profiling and quantification (by HPLC and HPTLC) and DPPH assay; photoprotective effect (ability to protect UVA-irradiated HaCaT cells) and antioxidant enzyme activity	[61]
Cannabinoids and terpenes	*Cannabis sativa* (medicinal cannabis) inflorescences	Decarboxylation step of the preliminarily dried and cut inflorescences	Olive oil	All medical *Cannabis* oils were prepared using drug-to-solvent ratio 1:10 (*w*/*v*); the first extraction method did not include a decarboxylation step and inflorescences after mixing with olive oil were kept in a water bath at 98 °C for 120 min; in the two subsequent extraction methods, decarboxylation of the plant material was carried out at 115 °C for 40 min and at 145 °C for 30 min (followed by a 3 min turbo-extraction before maceration), respectively.	HPLC-Electrospray Ionization-Tandem Mass Spectrometry (HPLC-ESI-MS/MS) analysis of the cannabinoids; GC-MS and GC-FID analysis of the terpenes	[66]
Cannabinoids	*Cannabis sativa* L. (Bedrocan^®^ and FM2)	Decarboxylation of acidic cannabinoids: plant material was heated in a petri dish put in an oven at 115° for 40 min; FM2 samples were also prepared at 125 °C for 40 min (effect the temperature). Unheated samples were used as control. Afterward, the extraction was carried out by maceration or ultrasonication	Olive oil	*Maceration*: 2 g of finely grinded Cannabis, decarboxylated or not, were added to 20 mL of olive oil and mixed. The mixture was stirred for 40 min and then immediately filtered to obtain the final oil. Preparation was also performed with 5 g of Cannabis and 50 mL of olive oil. (i.e., 5 g of sample in 50 mL of olive oil).	Analyzed in terms of volatile carbonylic organic compounds (CVCs), oxidized and conjugated fatty acids, tocopherol, and their oxidized forms. DSC analysis was also performed	[65]
Cannabinoids	*Cannabis sativa* (flowering tops)	Cannabis flowering tops were dried and grinded	Olive oil	A total of 500 mg cannabis flowering tops were placed in 5 mL olive oil and heated at about 98 °C for 120 min, cooled and filtered. For analysis of cannabinoids, 100 μL oil sample was extracted with 890 μL methanol: chloroform (9:1), sonicated 15 min and centrifuged at 3500× *g* for 5 min.	Analysis of cannabinoids by UHPLC-MS/MS	[58]
Capsaicinoids and volatile compounds	Chili pepper, dried (*Capsicum annuum*)	Ground and dried red-hot chili pepper product was purchased from a local market	Olive oil	Extractions were performed by using dried and ground chili peppers at two different concentrations (10% and 20% by weight); duration of the infusion process was 30 days but samples were taken also on the seventh and fifteenth day of infusion.	Quantification of capsaicin and dihydro-capsaicin by HPLC analysis and volatile compounds identified in microgram per kg olive oil by SPME coupled with GC-MS; activity toward ABTS radical cation	[68]
Essential oils and lipophilic antioxidants	Oregano (*Origanum vulgare* L.), rosemary (*Rosmarinus officinalis* L.) leaves and garlic bulbs (*Allium sativum* L.)	Dried spices were ground to pass 0.5 mm sieve and then purged with nitrogen	Oil mixture (virgin and refined olive oils)	Ground leaves (oregano and rosemary) were added to the oil at 2% (*w*/*w*), mixed thoroughly by shaking with a magnetic stirrer in the dark and under nitrogen for 30 min; 100 g of peeled garlic chopped in 500 mL oil for 24 h with periodic shaking.	Oxidative stability test and consumer acceptability tests	[72]
Total polar phenols, tocopherols and chlorophyll pigments	Oregano (*Origanum vulgare* L.) and rosemary (*Rosmarinus officinalis* L.) leaves	Ground leaves	Olive oil	Ground leaves (oregano and rosemary) were added to the oil at 5% (*w*/*w*), mixed thoroughly by shaking for: 24 h, 48 h and 72 h at 40 °C in the dark. The plant material was removed by filtration.	HPLC analysis of polar phenolic compounds and pigments; oil stability indices at 120 °C (OSI), photo-oxidation resistance and consumer acceptability	[73]
Essential oils and lipophilic antioxidants	The aerial parts of oregano (*Origanum vulgare* L.) and rosemary (*Rosmarinus officinalis* L.) and *Salvia trilobal* shoots	Ground plant	Olive oil	The ground aromatic plant (0.25 g) was added to the olive oil (5 mL) sample in a screw-capped glass tube and was vigorously agitated for 1 min and kept for one month in the dark at room temperature.	Profile of phenolic compounds in the hydro-methanolic extracts of the enriched olive oils; antiradical activity (DPPH assay)	[74]
Essential oils and lipophilic antioxidants	Rosemary (*Rosmarinus officinalis* L.), sage (*Salvia officinalis*) and thyme (*Thymus vulgaris*)	Dried spices were ground into fine powder to pass 60 mesh (0.250 mm) sieves	Sunflower oil	A total of 10 g of each herb was soaked in 40 g sunflower oil with continuous agitation at 50 °C for 24 h then allowed to stand and filtered.	Inhibition of conjugated dienes formation, reducing power, *beta*-carotene-linoleic acid assay	[81]
Volatile aroma compounds (borneol, camphor, o-cymene, eucalyptol, terpinen-4-ol, limonene and α-pinene) and non-volatile compounds (carnosol, carnosic and rosmarinic acid)	Rosemary (*Rosmarinus officinalis* L.) leaves	Dried rosemary leaves were provided by a producer after deodorization and then were mechanically ground and passed through No. 60 mesh screens (0.25 mm) for further maceration	Grapeseed, peanut, rapeseed, almond, avocado, soybean, wheat germ, sunflower, corn, olive, apricot and hazelnut oils	A total of 3.75 g of fine plant material powder was poured into flask(s) containing 25 mL of various vegetable oils on a magnetic stirrer plate at 40 °C for 3 h. After that all samples were centrifuged (at 2739× *g* for 15 min) at 4 °C in a refrigerated centrifuge.	Analysis of volatile, non-volatile antioxidant and total phenolic compounds by head space-solid-phase microextraction (SPME) coupled with GS-MS, HPLC-DAD and UV-VIS spectroscopy, respectively; solubility prediction by the sophisticated COSMO-RS simulation	[75]
Volatile aroma compounds VACs	Basil (*Ocimum basilicum* L.)	Dried spice was ground into fine powder to pass 60 mesh sieves (0.250 mm)	Refined sunflower, olive rapeseed, peanut and grapeseed	A portion (3.75 g) of ground plant material was poured into flask containing 25 mL oil on a RT—10 magnetic stirrer plate over 4 h in a temperature-controlled chamber.	Theoretical modeling of Hansen solubility parameter(s) HSP, analysis of volatile aroma compounds in aromatized oils	[76]
Essential oil (VACs)	Bitter orange (*Citrus aurantium* L.) peels	Peels were cut into small pieces	Refined and deodorized corn oil	Different quantities (5 g, 10 g or 15 g) of peels were homogenized in 40 mL oil at 20 °C and shaking speed 1000 rpm. Time intervals: 1 h, 2 h, 3 h.	Content (mg/mL oil) and percentage (%) of volatiles retained in deodorized oil	[82]
Total phenols, flavonoids, tannins, terpenoids, alkaloids, steroids and saponins	Moringa leaf (*Moringa Oliefera* Lamk.)	Moringa leaf powder was obtained directly from a producer	Virgin coconut oil and extra virgin olive oil	Moringa leaf powder was added to oil samples with varying percentages of the plant material (0, 10, 30 and 40%), heated at 50 °C for 2 h and finally extracts were filtered. Experiments with variations of temperature (50, 60 and 70 °C) during extraction, as well as heating time, (1, 2, 4 and 6 h) were also performed.	Total phenols and qualitative phytochemical screening	[77]
Lipid soluble antioxidants	Fifty-six (56) Algerian plant products including 17 green vegetables, 18 fresh fruits and 21 medicinal and aromatic herbs	Plant sources were washed with distilled water and peels were removed from the dried fruits; herbs were dried in the open air and only leaves were taken for the analysis, and from vegetables only pulp	Olive oil	About 10 g of olive oil was added to 1 g of homogenized plant material. The mixture was then heated at 50 °C with stirring for 0.5 h and centrifuged at 3000× *g* for 10 min. A total of 1 g of supernatant was dissolved in DMSO and the resulting solution was used for the further analysis.	Reducing power, i.e., RP and RSA (radical scavenging activity toward DPPH-radical)	[78]

**Table 2:** Intensification techniques (ultrasound, microwave, pulse electric field) applied for extraction of phytochemicals from natural (re)sources with edible oils.

Target Compounds	Natural Sources	Processes			Analysis	Ref.
		Sample Preparation	Oil Type	Extraction Conditions		
Carotenoids (9-*cis*-carotene, *alpha* and *beta*-carotene, lutein,)	Fresh carrots (85% water content) *Daucus carota*	Drying followed by shredding and grinding into fine powders using liquid nitrogen and sieving through 60 mesh (0.25 mm)	Sunflower oil	One liter of sunflower oil was placed into toughened glass tank and mixed with fresh shredded carrots, in varying ratios from 1:10 to 3:10, and then submitted to ultrasound-assisted extraction (UAE at a frequency of 20 kHz with different ultrasonic intensity) for 1 h.	Optimization of UAE (a sample of 1 mL was collected from the mixture during extraction process at every 10 min); UV-spectrophotometric and chromatographic (UPLC–DAD–MS) analysis of carotenoids	[13]
Carotenoids	Carrot residues	The residues were dried at 20 °C using a constant temperature electrothermal dryer with a fan until <1% moisture was reached, then were pulverized and sieved through 80 mesh	Cottonseed oil	A total of 10 g dried carrot residue powder was mixed with 50 mL oil (1:5) and subjected to ultrasonic frequency at 40 kHz. Different ultrasonic power (100–300 W), duration, i.e., extraction time and temperature (40–60 °C) were applied.	Color values; total carotenoid and phenolic content; identification and quantification of volatile compounds	[14]
Carotenoids	Carrot juice processing waste	Supplied by a producer; the by-product was freeze-dried and ground in a waring blender	Flaxseed oil	An amount of 25 mL of extraction vessel (88 mm height and 25 mm diameter with a silicone cap) was placed in microwave extraction device and the extraction process was operated after microwave power and extraction time were set up according to an experimental design (the temperature did not exceed 110 °C).	Total carotenoid content, quantification of *beta*-carotene by HPLC, color measurements	[15]
Carotenoids	Peach palm fruit (*Bactris gasipaes*) peel	Dry peel dispersed in 4 mL of soybean oil. Optimization of UAE (temperature, time and solid-solvent ratio)	Soybean oil	Optimal conditions for ultrasound-assisted extraction: 48 °C, extraction time of 28 min, solid-solvent ratio of 0.0037 g/mL.	Yield carotenoids (mg/100 g) ultrasound-assisted extraction (UAE) was compared (151 mg/100 g oil for 28 min) with two other extraction methods, i.e., Soxhlet method (169 mg/100 g oil for 6 h) and maceration (113 mg/100 g oil for 6 h)	[16]
Carotenoids	Mango pulp (*Mangifera indica*)	Blanching at 75 °C and addition of pectinase and cellulase followed by enzyme inactivation and cooling ≤ 10 °C	Flaxseed, groundnut and sunflower oil	Extraction with ultrasound, microwave (MW) and high shear dispersion (HSD) techniques.	Color values; total carotenoid content, antiradical activity (toward DPPH and ABTS) and FRAP assay	[17]
Carotenoids	Orange (*Citrus sinensis* L.) peels	The oranges, at the stage of commercial maturity, were washed with distilled water and the peels were processed in a grinder and stored at −20 °C	Olive oil and sunflower oil	A total of 1 g of fresh orange peels was mixed with 20 mL oil. UAE was applied for 20 min with ultrasound intensity of 60% (120 W) at 45 °C. The resulting mixture was filtered.	Color values; total carotenoid content, antioxidant capacity determined by TEAC and FRAP assay and antiradical (toward DPPH) activity	[18]
Carotenoids	Pomegranate (*Punica granatum* L.) peels	Dried ground and powdered pomegranate peels were mixed with 200 mL oil in a thermostat-controlled water bath for 10–60 min	Soybean and sunflower oil	UAE for 30 min; the extraction temperature (20–60 °C), the peels/solvent ratio varied from 1:10 to 3:10 and the amplitude level (20 and 60%) were optimized. Compared to classic extraction by organic solvents (hexane: isopropanol, 3:2).	Total carotenoid analysis (the optimum extraction yield was about 0.3255 mg carotenoids per 100 g of dry peels); comparison of the quality of untreated oil and the oil treated with ultrasound at the optimum (acid value, peroxide value, and conjugated dienes)	[19]
*Beta*-sitosterol and carotenoids	Sea (*Hippophae rhamnoides*) buckthorn pomace	Dried pomace consisting of peels and seeds was supplied by a Mongolian producer	Triacylglycerols TAG of refined oils (soybean and rapeseed) and medium-chain TAG	Samples of 2 g dried pomace were mixed with 20 mL oil (1:10) and stirred (at 25 °C) for 24 h. The suspensions were sonicated for one hour, then centrifuged at 9100× *g*, and finally supernatants were filtered (PTFE-0.45 μm).	Oil in water or O/W emulsion preparation, measurement of droplet size and size distribution; *beta*-sitosterol and carotenoid analysis	[20]
Lycopene	Tomato (*Solanum licopersicum*) wastes	Lyophilization (after the temperature reaches −80 °C, it is increased to −40 °C, at a pressure of 2 mBar); grinding	Grapeseed oil, olive, and peanut oil	A total of 10 g lyophilized tomato waste was added to a beaker containing 50 mL oil (1:5); high intensity ultrasound extraction was performed (20 kHz frequency, 80% amplitude) for 20 min pulsations of 40 s and intervals of 20 s.	Native oils (without lycopene) showed higher antiradical activity toward DPPH compared to the corresponding enriched ones	[29]
Phenolic compounds	Saffron (*Crocus sativus* L.) waste	Plant material was composed of *C. sativus* flowers	Sunflower oil	Phenolic content was extracted by oil using ultrasound at 35 kHz and the mixture was centrifuged at 1411 rcf for 10 min. Then the hydrophilic fraction of the enriched oil was prepared from 10 g extracted with 20 mL water-methanol (2:8)	The optimized conditions (solid-to-liquid ratio, temperature and duration) resulted in heightened phenolic content and antioxidant activity of the enriched oil	[44]
Curcuminoids	*Curcuma longa* L. rhizomes	Cleaned, sliced, and dried rhizomes of *Curcuma longa* L	Coconut oil, almond, castor oil, two olive oils, peanut, rice bran oil, two sesame oils, and two sunflower oils.	An amount of 2–6 g rhizomes treated with 20 g coconut oil, microwaved at 800 W for 0.5–1.5 min 1–3 times and then vacuum filtered.	Analyzed for individual and total curcuminoid content by HPLC	[53]
Curcuminoids, phenolic and polyphenolic compounds	*Curcuma longa* L. rhizomes		Extra virgin olive and virgin coconut oil	A total of 3 g of turmeric powder in 10 mL oil with variations of adding surfactant: 0 μg, 10 μg, 20 μg and 30 μg; in water bath sonicator (40 kHz) for different time intervals 10, 15, 20 or 25 min.	TPC, qualitative phytochemical screening and functional groups identification with FTIR	[52]
Cannabinoids and volatile compounds	*Cannabis sativa* L. inflorescences (Bedrocan^®^ and Bediol^®^ medical *Cannabis* chemo-types)	Grinding in a planetary mill with stainless balls (20 mm diameter) at a frequency 25 Hz to obtain fine powder; Decarboxylation step: in static oven at 145 °C for 30 min	Olive oil	Ultrasound extraction at a frequency 35 kHz for 30 min (without oil heating step); ratio Cannabis material/oil 1:10.	Oxidative stability of oily preparations from *Cannabis sativa* during storage	[62]
Cannabinoids	*Cannabis sativa* L. (Bedrocan^®^ and FM2)	Decarboxylation of acidic cannabinoids: plant material was heated in a petri dish put in an oven at 115 °C for 40 min; FM2 samples were also prepared at 125 °C for 40 min (effect the temperature)	Olive oil	A total of 2 g of finely grinded Cannabis, (decarboxylated or not) was dispersed in 20 mL oil (1:10) at 25–27 °C and the extraction was conducted with 2 mm sonotrode (S26d). For 20 mL sample different sonication times were used (10, 20 and 30 min) with amplitude of 60%.	Total content of carbonyl volatile compounds (CVCs), oxidized and conjugated fatty acids, determination of tocopherol and their oxidized forms and differential scanning calorimetry DSC of treated oils	[64]
Cannabinoids	*Cannabis sativa* L. (Bedrocan^®^ and FM2)	Decarboxylation of acidic cannabinoids: plant material was heated in a petri dish put in an oven at 115° for 40 min; FM2 samples were also prepared at 125 °C for 40 min (effect the temperature). Unheated samples were used as control. Afterward, the extraction was carried out by maceration or ultrasonication	Olive oil	Sonication: 2 g of finely grinded Cannabis, decarboxylated or not, were dispersed in 20 mL olive oil at room temperature and the extraction was conducted with 2 mm sonotrode (S26d). For the 20 mL sample, different sonication times were used (10, 20 and 30 min) with an amplitude of 60%. The same preparation was also performed increasing the solvent volume but maintaining the same ratio for grinded Cannabis and olive oil.	Analyzed in terms of volatile carbonylic organic compounds (CVCs), aldehydes and ketones originated from autoxidation of fatty acids; oxidized and conjugated fatty acids; and tocopherol and their oxidized forms. DSC analysis was also performed	[65]
Capsaicin, β-carotene, total phenols	Hot pepper (red) paste	Different amplitudes (40–80%), temperatures (30–60 °C) and times (5–20 min) were used with 20 g powder samples in 50 mL of olive oil. Compared to maceration (20 g sample extracted with 50 mL olive oil)	Olive oil	Ultrasound-assisted extraction (UAE) was applied in refined olive oil with a 0.4 solid: solvent ratio and 0.4 duty cycle with different parameters such as amplitude (40–80%), temperature (30–60 °C), and time (5–20 min).	The effects of these parameters on the β-carotene, capsaicin, total phenolic content, and antioxidant activity of the extracts were studied. UAE was found to be more advantageous than maceration because it produced higher amounts of extract in a shorter time	[70]
Capsacinoids	Dried red-hot chili pepper powder (*Capsicum annuum* L.)	Chili pepper-flavored olive oils (CPOOs) were prepared by infusion of chili pepper at concentrations 10% and 20% for 7 days maceration (for comparison)	Olive oil	Amounts of 10% and 20% dried chili pepper in olive oil were subjected to ultrasound-extraction for 10 or 20 min; for microwave extraction, olive oil samples were added with 20% chili powder and treated for 10, 30 or 60 s.	Capsaicinoids were quantified by HPLC-DAD directly in the flavored olive oil and antioxidant activity was evaluated by the ABTS+ method	[69]
Capsaicin, carotenoids, phenolic compounds	Cumari-do-Pará (*Capsicum chinense* Jacq.) peppers	Peppers were sanitized with a 200 mg/L sodium hypochlorite solution for 15 min and washed with distilled sterilized water, and the seeds and pulps were ground, freeze-dried in a LS300 freeze-dryer (Terroni, SP, BRA) at −55 °C under a vacuum pressure of 55–100 μHg for 4 days, and immediately stored at −18 °C until use	Soybean oil, Brazilian nuts (Bertholettia excelsa H.B.) oil, palm olein	Ultrasound-assisted extraction (UAE) was performed at 800 W and 20 kHz. The freeze-dried pepper (1.0 g) samples were placed in 15 mL capped plastic tubes and individually mixed with 5 mL of oils obtained from soybeans, Brazil nuts or palm olein, and sonicated for 60 min. Subsequently, the mixtures were centrifuged at 13,000× *g* for 20 min at 25 °C, and the supernatants, comprising the oily extract (OE), were separated for further analyses. Extraction parameters, mass: solvent ratios, and extraction times were established after preliminary tests	Proximate composition of the pepper extracts, vitamin C, total phenolics, total carotenoids, capsaicin, antioxidant activities by the ABTS radical scavenging and β-carotene/linoleic acid assays/ Comparison with organic solvents extraction: TPC were extracted by 70% aqueous acetone solution; capsaicin was extracted with an 80% aqueous ethanol solution; total carotenoids were extracted using two solvents, namely acetone for the initial extraction phase followed by petroleum ether. Antioxidant capacity—by 50% methanol and 70% acetone solutions	[71]
Essential oil and volatile compounds of basil leaves (eugenol and linalool)	Basil (*Ocinum basilicum* L.) leaves, freshly cut	Olive oil (1 L) was placed into the sono-extraction reactor and different amounts of basil leaves were added to the oil	Olive oil	1. Isolation of basil essential oil by steam distillation 2. Conventional aromatization by maceration. 3. In a sono-extraction reactor, made of double mantle into which cooling water circulate (to keep constant t°); the intensity of ultrasounds is about 1 W/cm^2^ with a frequency of 25 kHz; different ratios were used for the solid-liquid extraction.	Scanning electron microscopy of basil leaves before and after extraction; kinetics of eugenol and linalool extraction in olive oil; GC/MS profiles of aromatized oils	[79]
Essential oil and volatile organic compounds of oregano leaves	Dried oregano (*Origanum vulgare* L.) leaves	The enrichment procedure was based mainly on the immersion of dry oregano in the oil under different conditions in order to optimize the enrichment of phenolic (rosmarinic) acid from the plant in the oil.	Virgin olive oil	Comparison of methods: 1. Variable amounts of dry oregano (1, 2, 3.5, 5 and 7 g) were added to 100 mL of VOO. The mixture was stirred at either 400 or 700 r.p.m. at 37 ± 3 °C for 30 h. A total of 2. 5 g of dry oregano was added to 100 mL VOO, the mixture was sonicated for 15, 30, 45 or 60 min. 3. Equal amounts of dry oregano and VOO were combined and vertically stirred (1000 r.p.m.) for a maximum of 24 h. 4. Equal amounts of dry oregano and VOO were combined, sonicated and vertically stirred (1000 r.p.m.) for 15, 30, 45 or 60 min.	Rosmarinic, *o*-coumaric and vanillic acids were measured in olive oil before and after enrichment by the different methods, by capillary electrophoretic separation and UV detection	[80]
Polyphenolic compounds (flavonoids), carotenoids and chlorophylls	Garden sage (*Salvia officinalis*), lemon grass, myrtle (*Cymbopogon citratus*, and *Backhousia citriodora*), laurel (*Laurus nobilis*), common fennel (*Foeniculum vulgare*), Basil leaves (*Ocinum basilicum*), wild thyme (*Thymus serpyllum*) nettle leaves (*Urtica dioica*) summer savory (*Satureja hortensis*), and rosemary (*Rosmarinus officinalis*) powder or leaves	Oils were filtrated and dissolved in cyclohexane before UV-Vis measurements	Olive oil	Approximately 0.75 g dry plant samples were put in 5 g olive oil in 15 mL glass bottles and subjected to either conventional or ultrasound maceration, the last at intensity of ultrasounds < 1 W/cm^2^ and frequency of 40 kHz.	UV-VIS analysis detected significant amounts of aromatic content extracted in olive oil by applying ultrasounds for only few minutes, especially for *Salvia officinalis* powder	[83]

## 5. Conclusions

The use of edible vegetable oils as alternatives to organic solvents for the extraction of valuable biomolecules from plants, plant-based waste and by-products is in line with the principles of green chemistry. On the other hand, the fact that such an approach ensures concomitant intake of lipids and antioxidants cannot be ignored. In this review, along with the classical extraction techniques such as maceration and infusion, some intensification procedures including ultrasound or microwave treatment are presented and summarized as well. The focus is on the following extracted bioactive compounds with antioxidant properties: carotenoids, crocins, curcuminoids, cannabinoids, capsaicinoids and fractions from medicinal/aromatic plants. These ready-to-use extracts possess valuable physical, chemical and biological properties that determine their suitability for inclusion in the composition of a final product. Achieving higher amounts of antioxidants after extraction from different plant sources using vegetable oils is related to the problem of optimizing the extraction parameters (solid-to-oil ratio, temperature, extraction time and technique, etc.). The characterization and analysis of the obtained oily extracts is closely related to their specific application in the food or cosmetic industry. Despite all the advantages, disadvantages related to the stability of the fortified oils are discussed as well. More research efforts are needed for a future implementation of the oil-based extraction systems at an industrial scale including long-term stability, quality control, validation, standardization, regulatory evaluation and green metrics assessment.

## Data Availability

No new data were created or analyzed in this study. Data sharing is not applicable to this article.
